# Unravelling the skills and motivations of Magdalenian artists in the depths of Atxurra Cave (Northern Spain)

**DOI:** 10.1038/s41598-023-44520-w

**Published:** 2023-10-13

**Authors:** Diego Garate, Olivia Rivero, Joseba Rios-Garaizar, Mª Ángeles Medina-Alcaide, Martin Arriolabengoa, Iñaki Intxaurbe, Juan F. Ruiz-López, Ana Belén Marín-Arroyo, Juan Rofes, Paula García Bustos, Antonio Torres, Sergio Salazar

**Affiliations:** 1 Instituto Internacional de Investigaciones Prehistóricas de Cantabria (IIIPC), Universidad de Cantabria, 39005 Santander, Spain; 2 Departamento Prehistoria, Historia Antigua y Arqueología, Universidad de Salamanca, 37008 Salamanca, Spain; 3 Arkeologi Museoa, Calzadas de Mallona s/n, 48006 Bilbao, Spain; 4 CNRS, Ministère de la Culture, PACEA, UMR 5199, Université de Bordeaux, 33600 Pessac, France; 5 Departamento de Geología, Euskal Herriko Unibertsitatea/Universidad del País Vasco, 48940 Leioa, Spain; 6 Departamento de Historia, Universidad de Castilla La Mancha, 13071 Ciudad Real, Spain; 7 Evoadapta, Departamento de Ciencias Históricas, Universidad de Cantabria, 39005 Santander, Spain; 8 School of Archaeology, University of the Philippines Diliman, 1101 Quezon City, Philippines; 9 Archéozoologie, Archéobotanique: Sociétés, Pratiques et Environnements (AASPE, UMR 7209), CNRS/MNHN, 75005 Paris, France; 10 National Museum of the Philippines, 1000 Manila, Philippines; 11 Departament d’Història i Arqueologia, Universitat de Barcelona, Carrer de Montalegre 6, 08001 Barcelona, Spain

**Keywords:** Archaeology, Cultural evolution

## Abstract

Atxurra cave has a decorated assemblage composed of more than a hundred engraved animal depictions. All of them are located in deep parts of the cave and most of them are hidden in raised areas, away from the main path. The main sector is the “Ledge of the Horses”, located at 330 m from the entrance of the cave. It is a space of 12 m long and 1.5 m wide, elevated 4 m above the cave floor. This area includes almost fifty engraved and painted animals accompanied by a dozen flint tools, three fireplaces, and around one hundred charcoal fragments from torches. This extraordinary archaeological record allows us to value the complexity of the artistic production inside the caves during the Upper Palaeolithic. Our study has confirmed that there is planning prior to artistic production, both in terms of the iconographic aspects (themes, techniques, formats), its location (visibility, capacity), and the lighting systems. Furthermore, the data indicates the panel was decorated to be seen by third parties from different positions and was expressly illuminated for this purpose. This evidence supports the role of rock art as a visual communication system in Upper Palaeolithic societies.

## Introduction

One of the most difficult tasks in understanding Upper Paleolithic rock art is the study of the technology of the artists, due to the lack of related remains in the archaeological record. Usually, we only have the result of the artistic production, the artwork itself, and almost no evidence about the making process. This is due to the absence of the tools used and the waste generated in this artistic production, either because they were not deposited in situ or simply because they have not been preserved.

This is the reason why the iconography, techniques, and stylistic features of the Upper Paleolithic are the basis of the research and potentially valuable information about cultural-knowledge exchange networks^1–9^. Regarding this, some authors have previously pointed out the social function of rock art as a visual communication system^10–14^,although the exact and probably diverse cultural and/or religious motivations remain unknown^15–17^.

Traditionally there has been a poor interest in the close archaeological context of rock art and this evidence had not been comprehensively recorded or studied^18^. In this sense, caves with archaeological contexts related to rock art offer excellent opportunities to delve into new approaches. Knowing the social implications of all the activities related to artwork production is decisive to obtain a deep and objective understanding of Palaeolithic symbolic activities^19^. Consequently, the recreation of the whole operational chain of the artistic production has been only possible when an archaeological record adjacent to the rock art exists^20^.Moreover, the landscape context and emplacement patterns studies might shed light on whether the social function of these activities was related to an open (public, accessible) or closed (private, restricted) communication system^21–23^.

This comprehensive approach is possible when we have closed contexts where archaeological remains have been preserved in exceptional conditions, e.g., La Garma Cave^24^ in Spain or Tuc d’Audoubert^25^, Cussac^26^ and Chauvet^27^ caves in France. But even in very visited caves, sometimes open to tourism, it has been possible to find well-preserved evidence away from the main areas of transit, for example in Etxeberri Cave^28^ in France or in Nerja Cave^29,30^ in Spain. This is also the case of Atxurra, which has suffered from significant anthropic activity over the last century but, in spite of this, it contains an extraordinarily well-preserved archaeological context associated with one of the main decorated panels of the cave.

## Context: Atxurra cave (Northern Spain)

**Figure 1:**
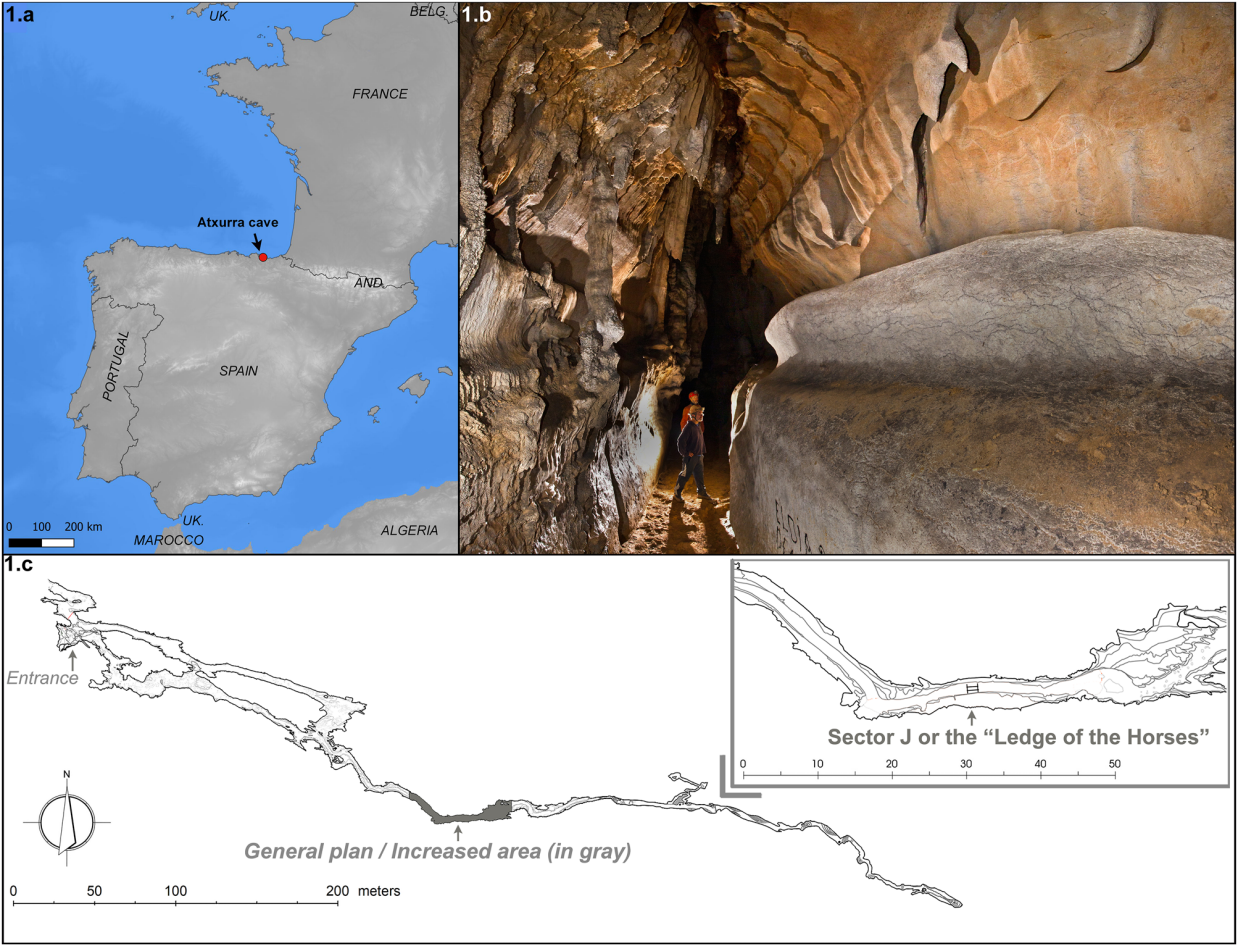
(**a**) Map showing the location of Atxurra Cave (Source: https://ec.europa.eu). (**b**) Photography of Sector J or the “Ledge of the Horses”, showing its location at the right wall of the passage (X. Gezuraga) (**c**) Plan of the cave generated from the scanned point cloud (Gim-Geomatics, SL) and caving survey (ADES).

The cave of Atxurra is a prehistoric site known since 1929^31^. It is formed in Aptian-Albian reef limestones (Lower Cretaceous) that emerge in the seaside of East Cantabrian region (Northern Spain) (Fig. 1a). The cave consists of two sub-horizontal conduits situated at different heights, the lower one called Armiña, and the upper one, Atxurra. The entrances are located on the right bank of the Zulueta stream, a tributary of the Lea River that flows into the Cantabrian Sea.

The natural gate used by prehistoric societies to access the cave system was the upper entrance of Atxurra^32,33^. It is currently 35 m above the stream, and it opens to a small vestibule where Gravettian, Magdalenian and Bronze occupation sequence have been identified^31,34^. There are two points of union between both levels, one of them a few meters from the upper-level occupation site and another at the end of the lower-level gallery of Armiña, under the point where the decorated sector begins. The upper level, where the rock art is placed, continues for around 600 m after this second connection (Fig. 1c).

In 2014, the archaeological excavations at the entrance of Atxurra were resumed to assess the potential of the remaining deposit. In 2015, during a systematic survey of the cave walls, D. Garate and I. Intxaurbe found several decorated panels with Magdalenian style engravings^35^.

The decorated sectors are between 186 and 366 m from the prehistoric access, and they are located mainly over lateral ledges that can be reached after climbing. In total, there are 22 decorated places in which more than a hundred animals, such as bison, horses, ibex, hinds, and aurochs, have been depicted. All of them are engraved and, in some cases, painted in black. The style and conventions suggest a Late Magdalenian (ca. 14,000–15,500 cal BP) attribution^36^. In other parts of the cave a few red stains, perhaps linked to the transit of people stained with ochre, have been also found^37^. In addition, all their adjacent floors contain archaeological remains (especially lithic tools and charcoals) abandoned during different visits in prehistoric times. Also, in the Armiña sector, a Late Magdalenian brief occupation with some lithics (including a firelighter), a fireplace, and an ochre stain were excavated and interpreted as an inner cave context^32^.

Sector J of Atxurra Cave, also called the “Ledge of the Horses”, is an elevated cornice at about 3.4 m high on the right wall looking from the entrance of the main gallery (Fig. 1b). The decorated side of the cornice is 12 m long, the overall width being 1.2 m and its useful surface (with a slope less than 30°) is 33 m^2^. It contains a panel with Palaeolithic art, with almost a hundred graphic units (GU), and several archaeological remains (lithic industry, combustion areas, and scattered charcoal remain from wooden torches) on the ground^36^. The sector is 329 m away from the entry, and to reach the platform, it is necessary to climb over an inclined slope located at the end of the cornice.

Concerning the accessibility and the reconstruction of the palaeo-geography of the cave, the geomorphological study combined with U/Th dating^33^, revealed that the whole passage, including the “Ledge of the Horses”, has not suffered significant morphological changes since its use by Magdalenian people. Only the floor of the passage that runs bellow the cornice would have been situated 15–30 cm lower due to an active horizontal flowstone and *gour* type formations since then.

## Results: reconstructing the artistic process in Atxurra Cave

A multiproxy approach to this archaeological space allows us to define, and recreate, all the operational chains involved in the artistic creation and use of the “Ledge of the Horses” in Atxurra Cave.

### Rock art iconography

**Figure 2:**
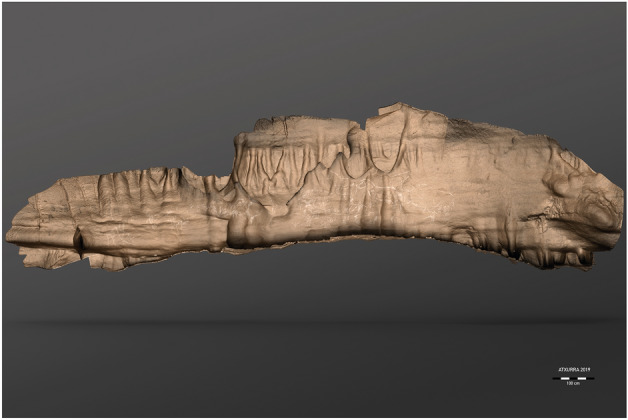
Reproduction of the rock art motifs from the “Ledge of the Horses” on the three-dimensional model (O. Rivero, J.F. Ruiz-López, I. Intxaurbe, S. Salazar, D. Garate). For the location of each figure, see Fig. 1*, *S2 of Supplementary Information S2.

The “Ledge of the Horses” contains the main concentration of rock art inside the cave. Eighty-five motifs are displayed on a surface of 9.68 m long and a maximum height of 2.42 m, from the extreme of the most opposed figures (Fig. 2). Forty-seven correspond to animals (19 ibex, 9 bison, 4 horses, 3 hinds, 1 deer and 11 indeterminate), 2 are defined as signs, and the rest as simple lines and stains (Supplementary Information S2). The panel is located on the right wall of the main gallery on top of a platform that presents a progressive inclination towards the viewer (between 50° and 64°), to the point that the narrow ledge does not allow to stand upright without holding onto the wall, so as not to fall into the void. The limestone surface presents slight sub-horizontal and vertical concave-convex undulations throughout its development.

On the right side of the panel, there is a high superposition between the motifs (Supplementary Information S3). Two detailed horses drawn by a mixture of scraped and incised engravings and black painting for one of them are surrounded by other animals (bison, hind, ibex), one over the others. On the central side, a dozen ibexes are distributed in the irregular morphology provided by the pendants, two of them in frontal view. Finally, on the left side of the panel, the motifs are more spaced with much fewer superpositions than on the right side, but we find the same themes (bison, horse, hind, and ibex).

A large number of ibex figures is noteworthy and relates the site with two Magdalenian ensembles in the west of Cantabrian Spain: El Bosque^38^ and Covarón^39^, but also with Niaux^40^ and Trois-Frères^41^ in the French Pyrenees. Furthermore, the panel contains some compositions found in other Upper Magdalenian caves, like the stylised hinds in a line present in Sovilla^42^ (central Cantabrian Spain) and Ker de Massat^43^ (central/eastern French Pyrenean).

The style of the animal figures allows their correlation with others located in different regional contexts^44^, helped by the fact that most of them have a complete format. The bison’s horns and legs perspective in two planes is usual in Middle/Upper Magdalenian chronologies in the Cantabrian, Pyrenees, and Périgord regions^45^. The horse figures display some particular conventions, like small lines representing the hair in the hindquarters, as in a portable figure from Linar Cave^46^ (Central Cantabrian Spain) and from El Polvorín Cave^47^ (Eastern Cantabrian Spain or the ventral M quartering, representing different tones of the pelage^48^. Equally noteworthy is the abundance of “wounded” animals with projectile-like linear motives and/or inverted “V” motifs inside them. Similarly, the presence of animals in frontal view -two ibexes- is relevant, as it is a convention with a very precise chronological attribution in portable art^44,49,50^.

Technically, engraving is almost exclusive, in different variants -incised, scraped, etc.-, sometimes combined with black paint (1 bison, 1 ibex and 1 horse, but very poorly preserved). In the animal figures, lines are formed by a series of engravings, and a single incision represents internal anatomical details (ears, eyes, etc.). Besides, some figures have scraped zones to depict internal quartering, and some hind figures have striated zones in the head and chest zone.

### Artistic technology

#### The tools kit

**Figure 3:**
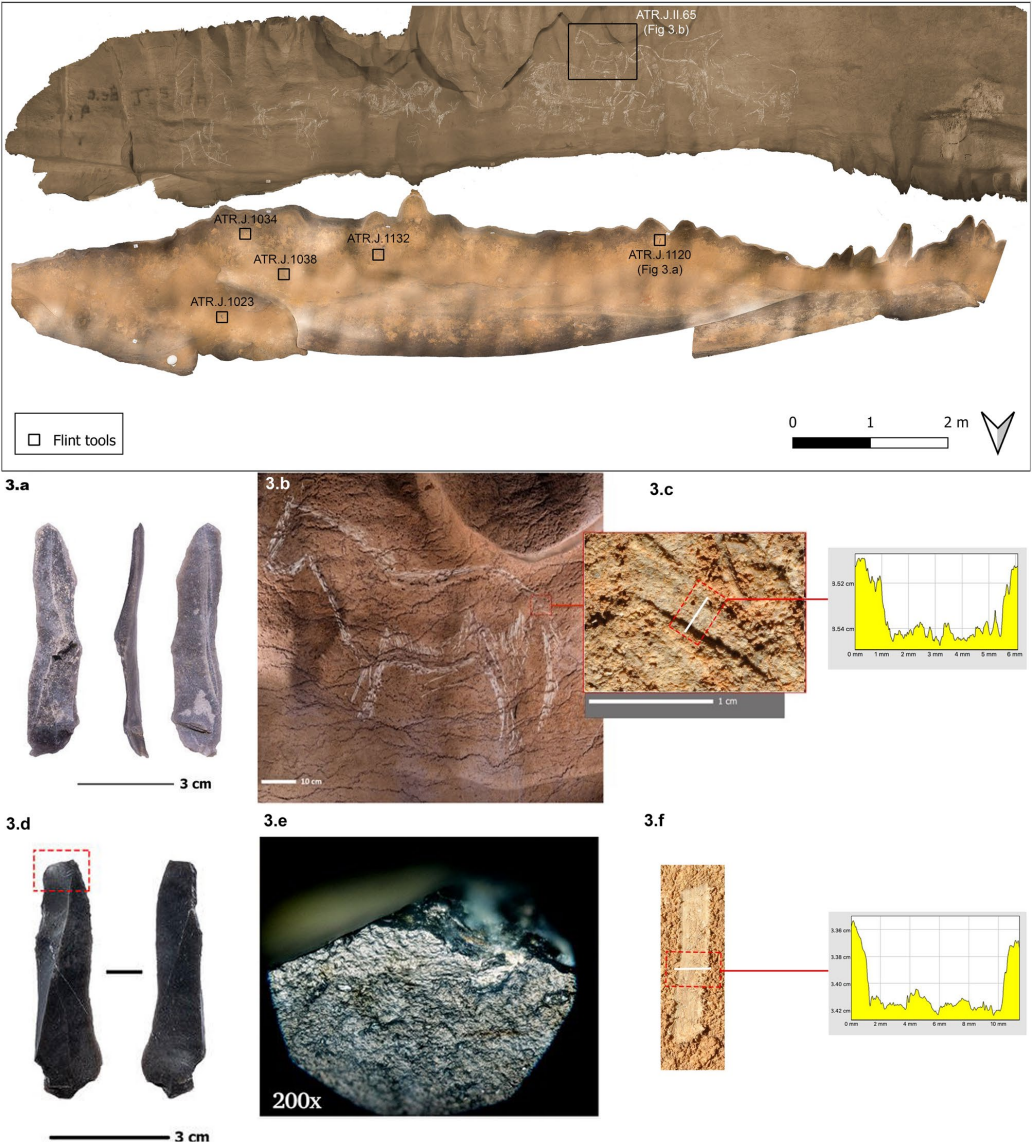
Georeferenced orthophotos of the floor and wall (rotated 180°) showing the location of the flint tools found on the surface: (**a**) Flint blade ATR.J.1120. (**b**) Engraved and painted in black horse ATR.J.II.65, found just above the last lithic tool. (**c**) Profile of an engraved trace of the last horse. (**d**) Replica of the flint blade ATR.J.1120. (**e**) Use-wear traces of the replica shows great similarity with the ones found in the archaeological tool (see supplementary information Fig. 2, S6). (**f**) Profile of an engraved trace made with the replica of the flint blade ATR.J.1120, showing great similarity with the archaeological one.

A total of 22 lithic artefacts were recovered in the initial surface survey (Fig. 3) and posterior excavation on the ledge (in addition to 31 macrofaunal remains and 58 small-mammals.). All of them are made on flint except for a Dufour bladelet made on lyddite. Among flint types, we have identified artifacts made on Flysch flint (N = 12), the most used flint type in the region^32,34,51^, north Pyrenean flint (N = 3), probably from Chalosse and the Pettit Pyrenees, translucent flint probably from Loza or Monte Picota (N = 1) and non-identifiable flint (N = 5). Among the materials there are five blades, two bladelets, one burin spall, one chunk and 13 chips (< 10 mm). Two of the blades are retouched (one dihedral burin and an inversely retouched blade), the two bladelets have inverse semi-abrupt retouch (Dufour bladelets), and the chunk probably formed part of a bec-like retouched tool.

Blades are regular, four of them wide, (between 19 and 28 mm). These wide blades are characteristic productions of the regional late Magdalenian and have been identified in the cave entrance occupation of Atxurra, in Armiña and in Abittaga^32,34,51^. These blades have been obtained through direct percussion with a soft hammer after careful preparation of the flaking surface and the cornice.

Interestingly, the burin spall does not refit with the single burin excavated at the site, and most of the chips cannot be related to the available pieces. These chips were likely produced during use-resharpening episodes, and therefore, we must interpret that some of the tools used for making the engravings were carried away by the artists.

Use-wear analysis has revealed use-related traces on the five blades. Traces are present in the pointed ends of the blades (2 active areas), in the trihedral formed by the confluence of an edge and a fracture plan (2 active areas), in the burin dihedral (1 active area), and in the lateral edges (3 active areas). All the identified traces are compatible with scraping, engraving, and incision of a medium-hard abrasive material (bright rounded polish, with abundant striations). The comparison with experimental traces obtained by engraving soft limestone reveals many similarities, therefore we can propose that all these pieces were used for preparing the panels (scraping with lateral edges), and for engraving. The narrow and acute trihedrals were used for engraving narrow and deep, V form, lines. The less acute distal ends and the burin dihedral were used for making wide and shallow lines with U form and with a characteristic “code-bar” surface.

#### The engraving process

Concerning engraving technology, an experimental program was developed to identify which tools were used for artistic purposes (Supplementary Information S4 and S6). To this end, the tools recovered from the ledge were replicated, and an experimental programme was developed based on making incisions in the Lamiñak cave, located in the same karst complex and with similar geological characteristics. The process has made it possible to identify different tools and active parts for the creation of different types of traces, which can be correlated with the graphic evidence on the panel^52^.

The evidence shows that the traces that make up the animal motifs and signs on the panel of the “Ledge of the Horses” present two main types of sections: V-section and flat section. The experimental reconstruction and statistical analysis of the profiles of archaeological and experimental traces allow us to determine that the flat sections were made with fractured ends of blades (which could correspond to tools ATR.1132 and 1120, as well as to the dihedrals of the burins in the case of piece ATR.1038, according to the data provided by the use-wear analysis).

In contrast, the V-shaped incisions could have been made either with the apex of a burin (ATR.1038) or with the pointed end of a blade (ATR.1023, 1132 or 1120). In all cases, the differences in the size of the incisions could be correlated with the difference in the size of the active parts of the tool.

Likewise, in some of the figures on the panel, there are traces of scraping (Fig. 3a,c,d,f) that could correspond to the use of the side of the blades, as evidenced by the traces of the use of the pieces (ATR.1038, ATR.1132).

These data can be correlated with the graphic construction of the figures. They show a predetermination of the type of active edge according to the type of stroke sought, which is in line with the different parts of the figures. For example, fur traces are often represented by flat profile incisions, while details such as eyes or ears are depicted with V-shaped profile incisions.

### Lighting systems

**Figure 4:**
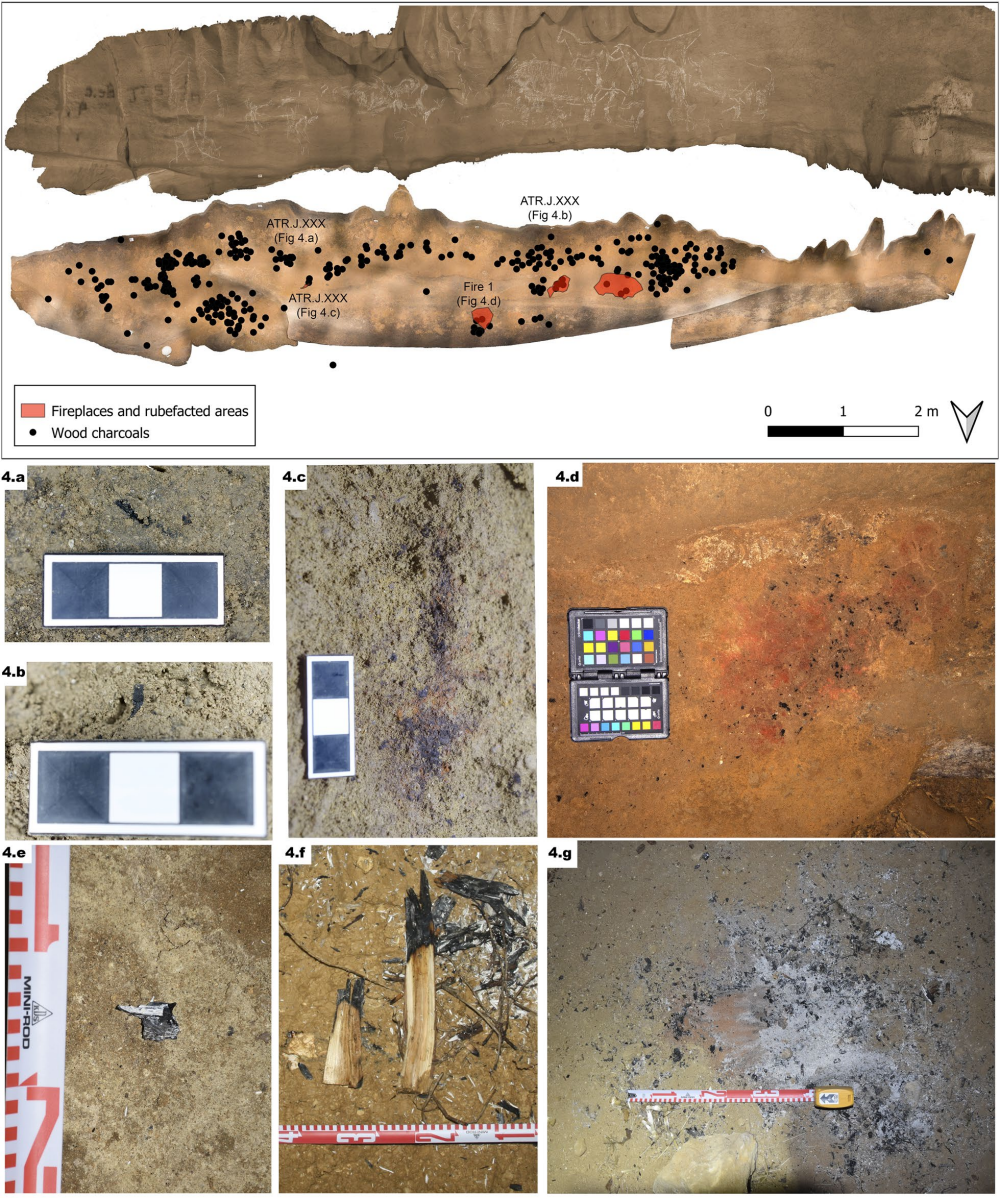
Georeferenced orthophotos of the floor and wall (rotated 180°) showing the location of the archaeological findings related to the lighting (wood charcoals and fireplaces and rubified areas): (**a**) and (**b**) Scattered charcoals ATR.J.1039 and ATR.J.1077 probably fallen from torches. (**c**) ATR.J.1125: A thick and elongated charcoal with rubified area in its borders on the left side of the panel, interpreted as a big branch falling down from a torch and burnt on the floor. (**d**) Fireplace 1 (accumulation of ash and micro-charcoals over a rubified ground). Experimental remains to be compared with the archaeological record: (**e**) Scattered charcoals fallen from torches. (**f**) A branch fell from a torch, burning over the surface creating an elongated burnt area. (**g**) Remains of a burnt fireplace: Accumulation of microcharcoals and ashes over a rubified ground surface.

Residues of two different prehistoric lighting sources have been found on the “Ledge of the Horses”: wooden torches and small fireplaces (Fig. 4). These have complementary luminous characteristics in the underground landscape. The torches facilitate mobile lighting, project light in all directions, they are easy to carry, and therefore they are appropriate for the transit and exploration of the cave. The hearths are stationary but release the upper limbs for the development of other activities (such as painting or engraving or just watching)^53^.

#### Torches

379 charcoals have been localised, and scattered on the floor (between surface level and 2.5 cm. deep) of the “Ledge of the Horses”. Generally, they are not associated with other combustion residues (ash, rubefaction clay…), as the charcoals inside the fireplaces, and are dispersed. 44.70% of them have been identified as the wood of juniper, 24.07% as deciduous oak, 9.52% as conifer, 7.67% as willow, 5.03% as angiosperm, 3.70% as indeterminate wood charcoal, 3.17% as scot pine, 1.32 as fir, 0.53% as indeterminate wood charcoal and 0.29% as not wood charcoal (Supplementary Information S7)*.* All these taxa are consistent with the paleoenvironmental data examined in the geographical and chronological context^54–56^. Also, most of them have been identified in the paleopolinic study carried out from sporadic occupation dated to the final Upper Magdalenian in the low gallery of Armiña^32^ and Atxurra^57^.

Our experimental endeavour confirm that the use of torches causes the gradual spill of the charcoals as the consumption progresses, increasing in number when the torch is oxygenated^53^. They are not normally associated with other combustion residues, which is why we associate these scattered residues with the use of woody torches (Fig. 4a,b,e). However, we have also located an elongated carbonized area with rubified borders on the left side of the panel, which we interpret as bigger torches residues (decimetric portion of branch) falling from a torch and burning in the floor (Fig. 4c,f) (Supplementary Information S8).

Juniper and deciduous oak charcoals have been identified in other similar contexts, linked to cave lighting with Palaeolithic art as the famous cave of Lascaux^58^, or in a closer context, in the caves of Morgota^59^, Praileaitz I^60^ and Ondaro^61^. Experimental analysis has confirmed that the addition of resinous elements to the fire (resinous wood for example), provides greater radiation in light intensity and duration than a fire exclusively fanned with hardwood^62^. In the other hand, willow wood is not a specially “good fuel” for lighting activity; however, it has high flexibility^63^. In this sense, it could have had a role in fastening or “braiding” the different wood elements of the torch^64^.

#### Fireplaces

Three rubefaction areas are located in the ledge (Fig. 4d,g). Micro-sedimentological analysis of two of them (F2, F3) shows a 1–2 mm thick ash deposit, placed over a rubified paleosurface indicating that they are in situ fireplaces. Additionally, poorly preserved microcharcoal, partially burned micro-faunal bones, and disperse animal fat have been found in the sediment. Underlying the sediment is a matrix-supported, compound of silt and fine sand particles of quartz, and 0.5–3 mm sized grains of lutite lithoclasts, sandstone lithoclasts, rip-up clasts, and anorthic ferruginous nodules.

Fourteen charcoals from these combustion structures have been identified (max. length 1.5 cm -although generally smaller-). In structure 1, two fragments of *Deciduous Quercus*, one fragment of *Juniperus* sp. and two indeterminate wood charcoals have been identified. In structure 2, four fragments of *Juniperus* sp., one fragment of *Deciduous Quercus* and one fragment of angiosperm compatible with *Quercus* sp. In Structure 3, one *Deciduous Quercus* and two wood charcoals have been identified. The anthropological study of the combustion structures suggests a joint use of juniper and oak wood, at least, observed in numbers 1 and 2. That is to say, the wood collected for the torches and the foyers could correspond to the same taxa, except for the remains of *Salix* sp. identified only in the scattered charcoals (related to the use of torches) interpreted as remains of the ligament to join the different parts of the same torch.

The palaeofloor of the “Ledge of the Horses” is made of host rock covered by 0 to 5 cm thick former fluviokarst deposit. In its micro-sedimentological analysis (*Supplementary Information S5*), an irregular darkened layer has been described 2 cm under the current surface (F1 sample) that could correspond to an accumulation of microcharcoals (Supplementary Information S8). Furthermore, dispersed, and concentrated ash remains have been found at different heights of the three samples (F1, F2 and F3), unrelated to hearths and deposited in a previous phase, which could also be related to the use of torches. At the top of the sequence, in situ, fireplaces have been described as a compound of rubifacted area under ash accumulations (F2 and F3 samples). Therefore, the micro-sedimentological study reveals at least two episodes that respond to two different types of lighting systems: 1. Visiting using torches, whereas charcoal and ash were dropped to the surface; 2. Visiting using fireplaces, where additionally footsteps deformed the sedimentary structure and could have buried the previous ash and charcoal remains. The study of the fauna recovered in the excavation of the sediment that covered a part of the ledge confirms these impressions, finding intermixed late-glacial species with current ones (Supplementary Information S9). There have hardly been sedimentary processes in it since the first human visit using torches, and these are mainly attributed to trampling and subsequent human passage.

### Visibility, capacity, and accessibility

We can describe the disposition of this rock art ensemble, classifying the figures in three thresholds according to the values of visibility of the natural shapes: low, medium, and high visibility. This is obtained by virtually simulating the scenario as if the fireplaces found under them were lit and counting the number of observers who can see each figure using GIS^23,65^ (Supplementary Information S4).

**Figure 5:**
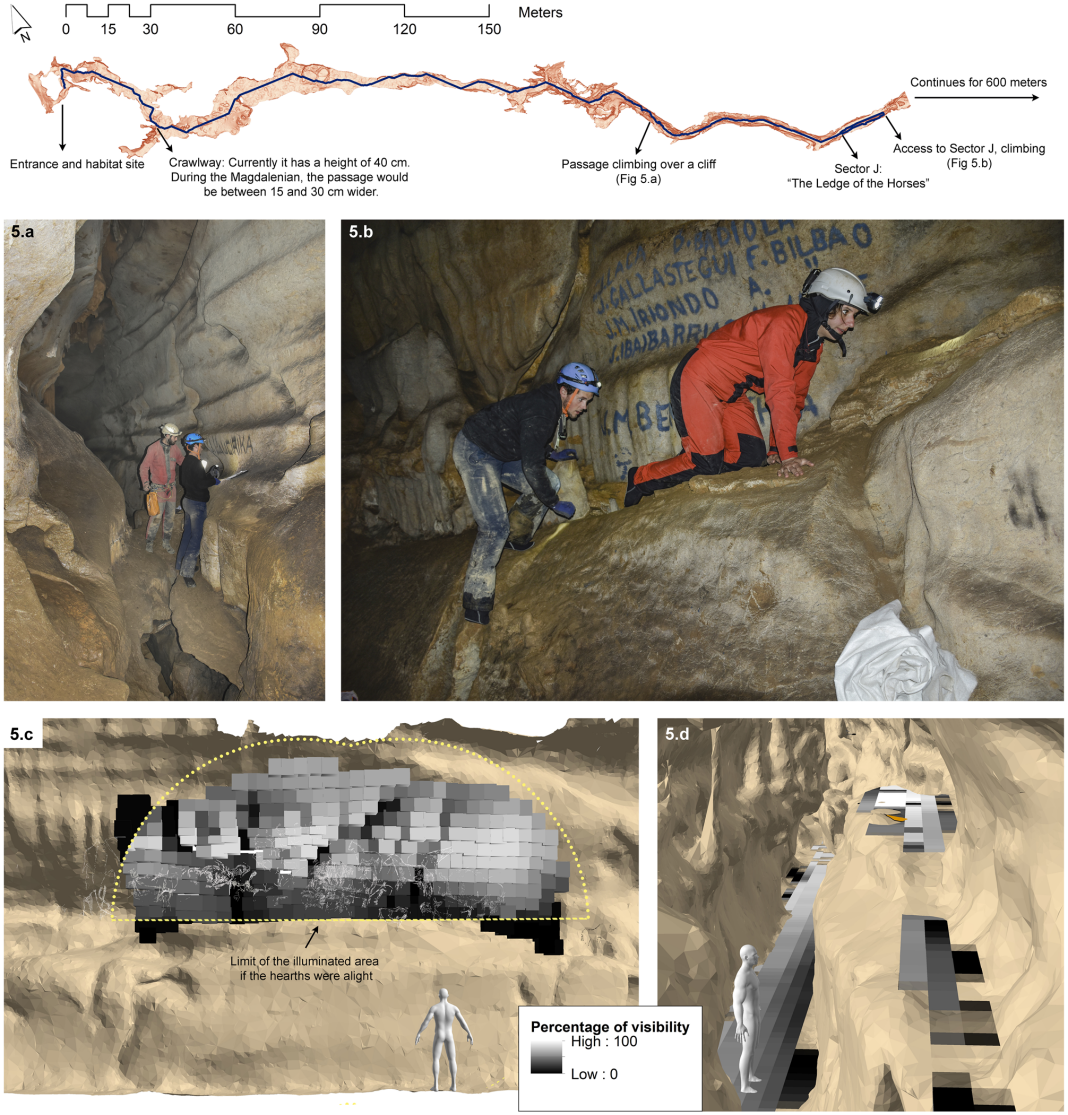
Digital Elevation Model (DEM) of the ground of the cave of Atxurra, showing the Least Cost Path (LCP) from the entrance used during the Prehistory to the “Ledge of the Horses”. It runs through the lower areas of the gallery, although there are some points where climbing is necessary (photos (**a**) -D. Garate- and (**b**) -I. Intxaurbe-). (**c**) Percentages of visibility of the decorated panel, showing the halo if the fireplaces were lit. (**d**) Percentages of visibility of the potential observers, showing that the points with the highest visibility are close to the opposite wall, or at the top of the shelf. The model presents the estimated average stature -1.60 m- for the European population during the end of the Upper Palaeolithic/Mesolithic.

The potential audience of this panel could have been located both above the ledge and in the lower part of the passage (Fig. 5c,d).

According to our analysis, the less visible figures (less than 33.33% of the total amount of observers could see this rock art) are those disposed in the lower part of the canvas, and in its corners. They are small, including some unfinished figures. In any case, there are also some striking examples of complex (combination of different techniques) and finished (abundance of corporal details like pelage) animal figures, like a small bison completely full of projectiles, or two hinds.

The medium threshold (between 33.33 and 66.66% of the observers) contains some of the biggest figures, like some bison (for example Atr.J.II.54) or parts of the biggest horse Atr.J.II.74 placed in the right part of the canvas.

The most visible graphic units of the panel (they could have been seen by between 66.66 and 100% of the observers) are in the upper places, especially a horse (Atr.J.II.65) and the head of another one (Atr.J.II.74) besides, a bison (Atr.J.II.80) located in the right, and a group of twelve ibexes located in an outstanding pendant in the centre of the panel. We need to highlight the case of the horses because they were displayed at very visible places and were made by combining several techniques (multiple lines, scraping, and black painting to emphasize the outline). It seems that these figures could have acted as an “eye-catching” of the rest of the panel.

On the other hand, some figures placed in the most visible parts of the wall are hardly perceptible because of the thinness of the engraving groove and the patina posed over it (like the big bison Atr.J.II.80 on the right). To this, the group of twelve ibexes would be added, because due to their small size, it must have been very difficult to see them from a distance, despite being in a prominent place on the panel. In fact, most of the figures is only perceptible from the ledge (Fig. 5c).

We need to point out that the three fireplaces were arranged strategically to illuminate almost the entire panel at the same time, perhaps as part of an activity that would allow the work to be shown in its entirety^53^. In any case, we must keep two things in mind. First, only one of the fires managed to be dated to a date consistent with that estimated for the graphic activity (see below), so we can only confirm that this element would illuminate the right side of the panel well. On the other hand, as the figures are located over 9.68 m of an irregular wall, it had to be impossible to see the entire panel at the same time from a single point.

Regarding the capacity, we have estimated that if potential observers carried hand torches, the illuminated area (both above and below the ledge) could gather about 18 people at a time. We want to emphasize that it is a maximum value, so if we measure a space in which potential observers have a flexibility of movements, we must consider enough lower capacity values (at least half of the obtained maximum value). The illuminated area if the fireplaces found at the top of the cornice were lit at the same time, would gather a maximum of 39 people at a time (or a half of this value -19- to stay comfortably), a considerably higher capacity when compared to other nearby caves of similar chronology^23^.

Finally, the accessibility analysis has estimated that the Least Cost Path (LCP) has a track distance of 357 m from the entrance. It presents some difficulties in the path -some crawling zones and small climbs- (Fig. 5a,b), which could be overcome with teamwork -without requiring specific technology or the need for extensive experience in caving- employing for it an estimated time of 39 min^22^.

### Bayesian analysis of the chronological sequence

Seven radiocarbon dates have been obtained from charcoal remains recovered on the floor of the ledge. four of them scattered wood from torches, another two placed inside the fireplace number 2, and the last one attached to the lithic tool ATR.J.1034. Concerning their taxonomy, two are *Deciduous Quercus*, another two *Juniperus sp.*, one *Salix sp.,* and two indeterminate wood fragments (Supplementary Information S7). Further sampling (14) has been tried specially inside the fireplace without positive results (low quantity of charcoal probably because of the complete combustion of the fireplaces).

The initial archaeological premise on which our Bayesian analysis is based on was that several phases of human occupation occurred during Prehistoric times in the interior of the cave. This hypothesis is supported by empirical observations of an archaeological and micro-sedimentological nature: a) On the outside of the cave there is an extensive archaeological sequence with various occupation levels^34^; b) On the “Ledge of the Horses”, at least two differentiated occupation levels have been observed on the floor of the ledge through the micro-sedimentological study.

**Figure 6:**
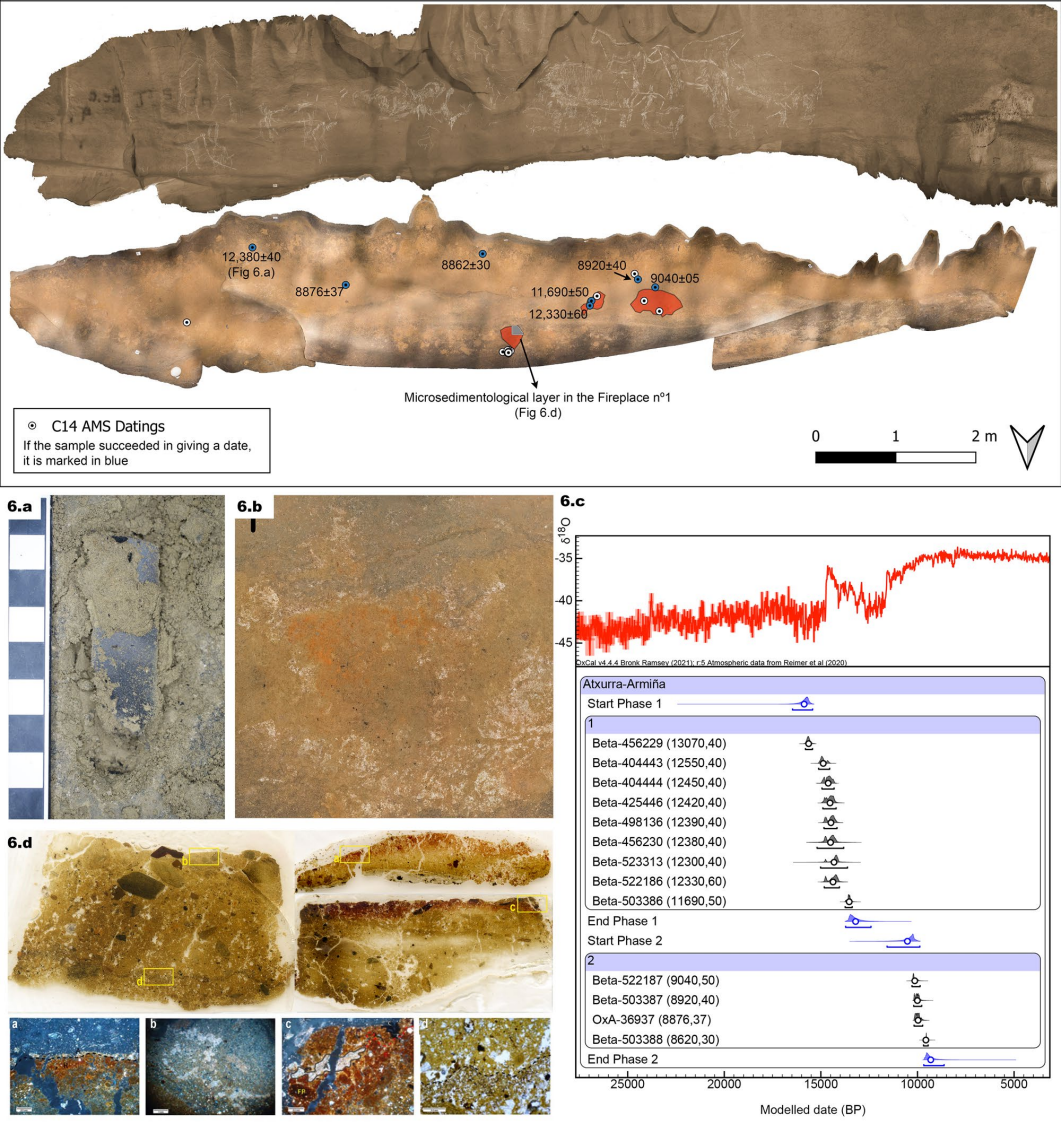
Georeferenced orthophotos of the floor and wall (rotated 180°) showing the location of the samples dated with C14 AMS): (**a**) Sample ATR.J.1034, with charcoal dated in 12,380 ± 40 uncal BP (lab reference) glued to a flint blade with use-wear traces compatible with a use for engraving limestone surfaces. (**b**) Fireplace nº 1, which was layered for micro sedimentological analysis. (**c**) “Multiple plot” representation of the Bayesian analysis performed in Oxcal 4.4. online, using IntCal20 dataset, for the characterization of the frequentation phases of Atxurra cave, as a climatic reference the GISP2 δ18O curve is included. (**d**) Thin sheets from the micro sedimentological analysis. The different microfacies referred in the text can be observed. For more detailed images consult supplementary materials.

The Bayesian inference points to 2 discontinuous phases between 15,429 and 8622 cal. BP, determined as highly probable (agreement index of 92,2 Overall) (Fig. 6). In the analysis we have also included the dates from the outermost deposits of the cave (Armiña and Atxurra), with the intention of providing the Bayesian model with greater precision for the comprehensive knowledge of the frequentation inside the caves. The first one is related to the Upper Magdalenian between 15,429 and 12,408 cal. BP and is composed of the fireplace number 2 and the *Juniperus* fragment added to a lithic tool. At the same time, this first phase of human activity is chronologically compatible with the sporadic occupation in the lower gallery of Armiña where we find similar stone tools^32^ and with the outside habitat of the entrance of the cave (level II)^57^. The second one is linked to the Mesolithic between 9882 and 8622 cal. BP and it is composed of the four charcoals dispersed over the ledge from *Deciduous Quercus*, *Salix* sp. and *Juniperus* sp. wood. No similar dates are recorded in the two habitat areas of Atxurra and in the sector with the sporadic occupation of Armiña. Between the two visits, the cave has been unoccupied for 3520 years. We also carried out the same Bayesian analysis, raising the possibility of the existence of three different phases of occupation. However, the agreement rate is higher for 2 phases (Supplementary Information S7).

This is to say, there is one frequentation in the “Ledge of the Horses” attributed to the engraving process -certified by the charcoal adhered to one of the engraving tools and the stylistic consistency of the engravings- and, at least, one fireplace was fired to contemplate the art. After a 3520 years interval of absence of subterranean visits, there is a second phase related to Mesolithic visitors. The dates correspond to torch charcoals and probably these people observed the engravings on the wall. Stylistically, there is no rock art that could be assigned to Mesolithic groups in Atxurra, but there are other examples of visits to decorated areas of other caves with Palaeolithic art (Bédeilhac, Rouffignac, Réseau Clastres, Lascaux, etc.)^18^.

## Discussion

In general, in the deeper contexts of the caves, the amount of archaeological remains is scarce. This is because they come from one-off activities and brief incursions, and these remains can be described accordingly as a ‘discrete’ archaeological context. The remains generally appear on the surface, on the ground or on the walls, where materials of different chronologies can be found, which do not necessarily belong to the same temporal episode.

Moreover, the remains are part of complex endokarstic taphonomic processes that make it difficult to identify them in situ, such as the covering by calcite flows, the subsidence of palaeosoils, sedimentation processes or variations in visibility due to fluctuations in the humidity index of the cave, to name but a few. The elucidation of this context is greatly facilitated by the geomorphological study of the site describing the transformations it has undergone over time^66^. In addition to all this, preservation is conditioned by surface exposure and uncontrolled visits by animals and humans—including during past tourist operations—prior to recent systematic surveys. Therefore, a specific methodology for this subterranean archaeology is needed^67^.The anthropic actions detected in these contexts generate different types of remains in the cavities^68^: thermal treatments, imprints, natural karstic environment transformation (stalactite and stalagmite stacking, structure building, breakage of stalactites, flowstones, etc.), procurement of raw materials (ochre, clay, moonmilch, etc.), deposits of different materials (bones, lithic tools, ornaments, etc.) in natural cracks, holes and surfaces of the cave, in addition of dyes (paint) or modifications of the support through engraving.

Thus, it is necessary to ensure the temporal coherence of these anthropic actions in the subterranean space by using the same type of analysis that is used for excavation stratigraphies: combination of dating techniques, refits between objects made in situ, taphonomic analyses and understanding of post-depositional processes…^69^. That is, we can identify events, even specific scenes, developed within the decorated caves at a given time. This is known as the ‘Pompeii syndrome’^70^. In contrast, in the case of the excavation of long stratigraphic sequences in the cave entrance habitats, it is almost impossible to dissociate or individualise the punctual activities carried out in different times.

In this way, it is possible to reconstruct human behaviour (underground progression, artistic process, tool production, ritual practice, etc.) within these symbolic spaces and to locate them in time.

This specific analysis is usually more common in caves that have remained sealed or isolated since the Paleolithic, such as Chauvet^27^, Cussac^26^, La Garma^24^ or Cosquer^71^, so it is surprising that a cave so deteriorated due to misuse for years like Atxurra keeps a context like this. The selection by the Upper Magdalenian societies of an elevated space and away from the most travelled path of the cave (which runs below the ledge) has allowed this particularity.

The comprehensive study through a multidisciplinary approach in the exceptional archaeological record of the “Ledge of the Horses” in Atxurra Cave, has provided us with relevant information about how the Palaeolithic human groups decorated their caves.

We have been able to correlate rock art with the immediate archaeological context. By means of an experimental programme and a use-wear analysis, we have analysed the technology of the engravings and compared them with the lithic pieces recovered from the “Ledge of the Horses”. This has allowed us to establish a direct relationship between the types of incised grooves and the specific tools used to make them. Together with the charcoal adhered to one of the flint blades, this demonstrates that the archaeological remains are part of the same action that took place at a specific time. This is to say, there is a synchrony between the different archaeological remains. Specially, we had established a synchronic phase between them, corresponding to the making and observation of the decorated panel *circa* 14,800 to 14,200 cal BP, this is to say during the local Upper Magdalenian. At the same time, we have understood how the artist used each tool type for specific graphic purposes, outlining the animals with one tool type and tracing the inner anatomical details with a different one. The tool kit has been prepared in advance and a selective process is evident, depending on the different kinds of tracings to be made.

The study of the charcoal remains has allowed us to reproduce the lighting systems used by the artists to access 300 m inside the cave and decorate a 10-m panel located 4 m high. Torches were used during art production and fireplaces were lit up after to behold the artworks, using oak and juniper wood (Willow wood, according to the radiocarbon results, seems to correspond to the Mesolithic phase). The characteristics of the lighting systems (torches and fires) imply a specific strategy for the management of smoke, the duration of the fuel or its location (not possible to engrave and light the fires at the same time), as shown by experimental archaeology^64^. Moreover, the spatial analysis of the figures inside the subterranean landscape and their relationship with the engraving and lighting techniques leads us to infer that the wall decoration was designed to be seen by a third party from the lower part of the gallery^65^. This is to say, this is evidence of the use of rock art from the Ledge of the Horses in Atxurra Cave as a visual communication system.

All these data indicate that the graphic activity in this sector of the cave presents a considerable degree of complexity, and, at the same time, requires an arduous process of prior planning, both in logistical or technical aspects (access, lighting, tools, postures, etc.) and in conceptual or social aspects (organisation of the panel, figures sizes and formats, etc.). This is to say, we can infer that these activities involved a significant investment of human, material, and time resources that the human groups had to spend and, therefore, their importance within these societies, far away from simplistic explanations of the phenomena proposed from the beginning of rock art research^72^.

## Conclusion

Through this study, we have proved that the parietal art decoration process featuring the Upper Magdalenian artist is the result of a complex and close relationship between the location of the artworks, the engraving techniques and technology, their visibility and organization in the space, and the illumination systems.

Moreover, it is also surprising to note that the iconographic display has been conceived and adapted to be seen by a third person and from another point in the gallery where the capacity is higher. This requires deep and thoughtful planning including an attentive observation of space, which allows us to emphasize the idea of so-called Paleolithic art as a system of visual communication with a specific message shared by the human group involved in the action or even by other different groups.

Consequently, the archaeological data recovered and analyzed in the “Ledge of the Horses” of Atxurra Cave is of relevance for a deeper understanding of one of the most relevant activities carried out by Upper Palaeolithic human groups.

**Figure 7:**
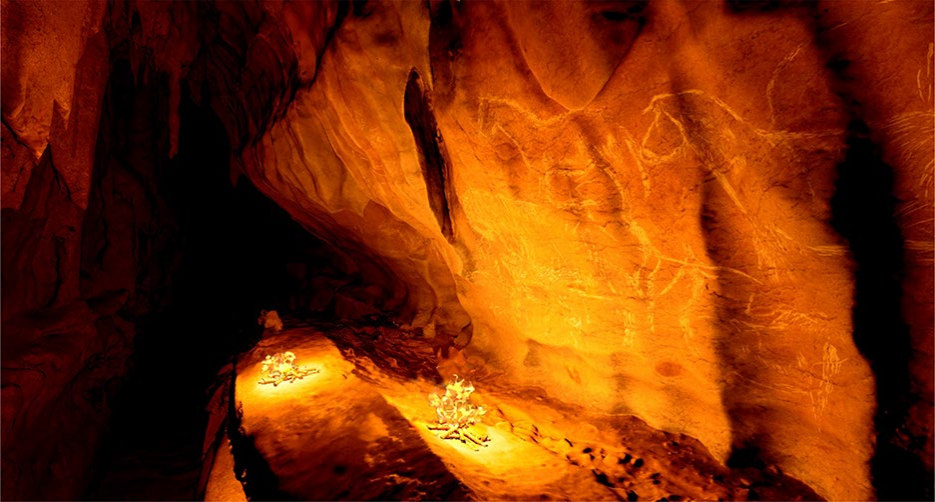
Virtual reality reconstruction of the ‘Ledge of the Horses” using the illumination from the fireplaces.

As a final step of our study, thanks to this comprehensive analysis and interpretation of the archaeological data, we have been able to virtually reconstruct all the components related to the scenario of the artistic process that took place^73^ (Fig. 7) (*Supplementary Information S10*).

### Supplementary Information

Supplementary Information.

## Data Availability

The datasets used and/or analysed during the current study are available from the corresponding author on reasonable request.
