# Supplementary material for: Unravelling the skills and motivations of Magdalenian artists in the depths of Atxurra Cave (Northern Spain)
Source: Sci Rep. 2023 Oct 13;13:17340. doi: 10.1038/s41598-023-44520-w (PMC10575969; doi:10.1038/s41598-023-44520-w)
Supplement: Supplementary file 1 — Supplementary Information. [file 41598_2023_44520_MOESM1_ESM.docx]

**Supplementary Information**

***S1: Rock art documentation techniques.***

***S2: Inventory of the rock art.***

***S3: Rock art stratigraphy.***

***S4: Rock art analyses.***

***S5: Survey and excavation techniques.***

***S6: Lithic analyses.***

***S7: Charcoal analyses.***

***S8: Hearth analyses.***

***S9: Zooarchaeological analysis.***

***S10: Virtual recreation.***

***S11: References in the Supplementary Information.***

***S1: Rock art documentation techniques.***

The imagery of Sector J in Atxurra Cave was mainly registered with close-range photogrammetry techniques. This was a serious challenge because of the complicated topography of this decorated area of the cave. It was necessary to find *ad hoc* solutions for two factors; on the one hand, the narrow ledge, scattered with important archaeological remains, posed a serious problem for positioning tripods and illumination systems; on the other hand, the wavy wall prevented from using the same light positions for the lengthy decorated panel. As described elsewhere (Rivero *et al*., 2019), we used a dual approach to face these challenges, in which every decorated sector was illuminated with raking flashlights to enhance the visibility of the myriad of incised hairline engravings, while the whole panel was recorded from scaffolding with even light.

Photogrammetric processing was executed in *Agisoft^©^ Metashape Pro*. Processing was carried out independently for every engraved sector, obtaining a high-resolution photographic texture that was used as the base for tracing motifs. This task was performed in situ on a *Wacom Mobile Studio Pro* that runs *Adobe^©^ Photoshop CC*. We considered that drawing on-site was the best option given the intricacy and complex superimpositions between figures in this panel. Once completed tracings were saved as .psd files and transferred to the photographic texture of the whole “Ledge of the horses”, where they were fitted into their right position thanks to the references that we have introduced in every 3D model during the recording process.

The result was finally rendered in *Adobe^©^ Photoshop CC*, using the 3D option of this software. As with any other 3D modelling software, it is highly customizable for texturing, illumination, settings, or camera positioning. Most of our renderings were based on orthographic views of specific areas of the panel, focused on some of the remarkable figures, with an illumination angle between 45º and 30º. Alternatively, some areas were rendered in oblique angles to stress the connections between imagery and the physiography of the bedrock.

***S2: Inventory of the rock art.***

| **CODE** | | **THEME** | | **TECHNIQUE** | **COORDINATES*** | | |
| --- | --- | --- | --- | --- | --- | --- | --- |
| **TU*** | **CU*** | **GU*** |  |  | **X** | **Y** | **Z** |
| J | I | 1 | Bison | Black Painting | 541299,808 | 4797025,03 | 73,834 |
| J | II | 1 | Line | Engraving | 541311,719 | 4797025,67 | 74,684 |
| J | II | 2 | Ibex | Engraving | 541310,889 | 4797025,05 | 74,074 |
| J | II | 3 | Ibex | Engraving | 541310,849 | 4797024,92 | 74,154 |
| J | II | 4 | Bison | Engraving | 541310,669 | 4797024,89 | 74,174 |
| J | II | 5 | Line | Engraving | 541310,599 | 4797024,94 | 74,144 |
| J | II | 6 | Ibex | Engraving | 541309,988 | 4797025,94 | 75,124 |
| J | II | 7 | Line | Engraving | 541309,938 | 4797025,53 | 74,764 |
| J | II | 8 | Ibex | Engraving | 541309,648 | 4797025,84 | 75,034 |
| J | II | 9 | Bison | Engraving | 541309,438 | 4797025,41 | 74,734 |
| J | II | 10 | Animal | Engraving | 541309,308 | 4797025,57 | 74,814 |
| J | II | 11 | Line | Engraving | 541310,178 | 4797025,22 | 74,474 |
| J | II | 12 | Line | Engraving | 541310,158 | 4797025,04 | 74,134 |
| J | II | 13 | Hind | Engraving | 541309,878 | 4797025,07 | 74,354 |
| J | II | 14 | Line | Engraving | 541309,958 | 4797025,31 | 74,614 |
| J | II | 15 | Sign | Engraving | 541309,748 | 4797025,12 | 74,444 |
| J | II | 16 | Animal | Engraving | 541309,538 | 4797024,96 | 74,294 |
| J | II | 17 | Hind | Engraving | 541309,138 | 4797025,21 | 74,514 |
| J | II | 18 | Deer | Engraving | 541308,738 | 4797025,18 | 74,514 |
| J | II | 19 | Bison | Engraving | 541308,588 | 4797025,29 | 74,604 |
| J | II | 20 | Animal | Engraving | 541308,538 | 4797025,19 | 74,534 |
| J | II | 21 | Line | Engraving | 541308,178 | 4797025,53 | 74,744 |
| J | II | 22 | Animal | Engraving | 541308,308 | 4797025,04 | 74,274 |
| J | II | 23 | Bison | Engraving | 541307,628 | 4797025,37 | 74,814 |
| J | II | 24 | Animal | Engraving | 541307,448 | 4797025,29 | 74,754 |
| J | II | 25 | Line | Engraving | 541307,168 | 4797025,37 | 74,944 |
| J | II | 26 | Line | Engraving | 541307,908 | 4797025,28 | 74,544 |
| J | II | 27 | Line | Engraving | 541306,948 | 4797025,11 | 74,774 |
| J | II | 28 | Bison | Engraving and black painting | 541306,998 | 4797025,33 | 74,974 |
| J | II | 29 | Horse | Engraving | 541306,948 | 4797025,01 | 74,564 |
| J | II | 30 | Animal | Engraving | 541307,038 | 4797024,96 | 74,434 |
| J | II | 31 | Animal | Engraving | 541306,488 | 4797025,25 | 74,624 |
| J | II | 32 | Line | Engraving | 541306,548 | 4797025,35 | 74,814 |
| J | II | 33 | Ibex | Engraving | 541306,508 | 4797025,77 | 75,014 |
| J | II | 34 | Ibex | Engraving | 541306,568 | 4797025,99 | 75,264 |
| J | II | 35 | Ibex | Engraving | 541306,378 | 4797026,05 | 75,414 |
| J | II | 36 | Line | Engraving | 541306,468 | 4797025,67 | 74,944 |
| J | II | 37 | Line | Engraving | 541306,168 | 4797025,43 | 74,614 |
| J | II | 38 | Line | Engraving | 541306,168 | 4797025,56 | 74,854 |
| J | II | 39 | Ibex | Engraving | 541306,308 | 4797025,9 | 75,224 |
| J | II | 40 | Ibex | Engraving | 541306,148 | 4797026,15 | 75,464 |
| J | II | 41 | Ibex | Engraving | 541306,158 | 4797026,02 | 75,304 |
| J | II | 42 | Ibex | Engraving | 541305,928 | 4797025,8 | 74,984 |
| J | II | 43 | Line | Engraving | 541305,798 | 4797025,69 | 74,884 |
| J | II | 44 | Ibex | Engraving | 541305,838 | 4797026,06 | 75,304 |
| J | II | 45 | Line | Engraving | 541305,778 | 4797026,26 | 75,564 |
| J | II | 46 | Ibex | Engraving | 541305,858 | 4797026,19 | 75,494 |
| J | II | 47 | Ibex | Engraving | 541305,638 | 4797026,11 | 75,434 |
| J | II | 48 | Ibex | Engraving | 541305,648 | 4797026,22 | 75,584 |
| J | II | 49 | Ibex | Engraving | 541305,318 | 4797026,14 | 75,514 |
| J | II | 50 | Line | Engraving | 541305,978 | 4797025,51 | 74,744 |
| J | II | 51 | Line | Engraving | 541306,118 | 4797025,31 | 74,474 |
| J | II | 52 | Line | Engraving | 541305,528 | 4797025,5 | 74,764 |
| J | II | 53 | Bison | Engraving | 541305,458 | 4797025,38 | 74,504 |
| J | II | 54 | Bison | Engraving | 541304,718 | 4797025,56 | 74,834 |
| J | II | 55 | Line | Engraving | 541305,178 | 4797025,96 | 75,374 |
| J | II | 56 | Line | Engraving | 541305,438 | 4797025,41 | 74,594 |
| J | II | 57 | Animal | Engraving | 541305,158 | 4797025,59 | 74,864 |
| J | II | 58 | Line | Engraving | 541304,898 | 4797025,56 | 74,824 |
| J | II | 59 | Bison | Engraving | 541305,188 | 4797025,38 | 74,464 |
| J | II | 60 | Line | Engraving | 541305,305 | 4797025,54 | 74,85 |
| J | II | 61 | Line | Engraving | 541305,136 | 4797025,45 | 74,663 |
| J | II | 62 | Line | Engraving | 541304,638 | 4797025,39 | 74,624 |
| J | II | 63 | Line | Black Painting | 541304,968 | 4797025,75 | 75,074 |
| J | II | 64 | Line | Engraving | 541305,218 | 4797025,45 | 74,614 |
| J | II | 65 | Horse | Engraving and black painting | 541304,458 | 4797025,73 | 75,224 |
| J | II | 66 | Ibex | Engraving | 541304,468 | 4797025,66 | 75,104 |
| J | II | 67 | Line | Black Painting | 541304,388 | 4797025,65 | 75,094 |
| J | II | 68 | Ibex | Engraving and black painting | 541304,278 | 4797025,35 | 74,704 |
| J | II | 69 | Line | Engraving | 541304,168 | 4797025,46 | 74,844 |
| J | II | 70 | Hind | Engraving | 541304,278 | 4797025,32 | 74,594 |
| J | II | 71 | Horse | Engraving | 541303,908 | 4797025,17 | 74,464 |
| J | II | 72 | Line | Engraving | 541304,148 | 4797026 | 75,474 |
| J | II | 73 | Line | Black Painting | 541304,338 | 4797026,03 | 75,524 |
| J | II | 74 | Horse | Engraving | 541303,618 | 4797025,58 | 74,924 |
| J | II | 75 | Line | Engraving | 541304,038 | 4797025,54 | 74,994 |
| J | II | 76 | Animal | Engraving | 541303,228 | 4797025,63 | 75,214 |
| J | II | 77 | Line | Engraving | 541303,778 | 4797025,28 | 74,614 |
| J | II | 78 | Line | Engraving | 541302,568 | 4797025,71 | 75,114 |
| J | II | 79 | Line | Engraving | 541302,168 | 4797025,67 | 74,964 |
| J | II | 80 | Bison | Engraving | 541302,368 | 4797025,54 | 74,764 |
| J | II | 81 | Animal | Engraving | 541302,478 | 4797025,4 | 74,404 |
| J | II | 82 | Sign | Engraving | 541302,068 | 4797025,42 | 74,264 |
| J | II | 83 | Animal | Engraving | 541303,208 | 4797025,2 | 74,414 |
| J | II | 84 | Ibex | Engraving | 541302,928 | 4797025,25 | 74,294 |
| J | II | 85 | Stain | Red Painting | 541303,968 | 4797025,52 | 74,954 |

***Table 1, S2* – *Inventory of the graphic units (GUs) of sector J****.* *TU: Topographic Unit (Sector). *CU: Compositive Unit (Panel). *GU: Graphic Unit (Motif). *Coordinates datum: ETRS 89 (UTM ZONE 30 N).

**
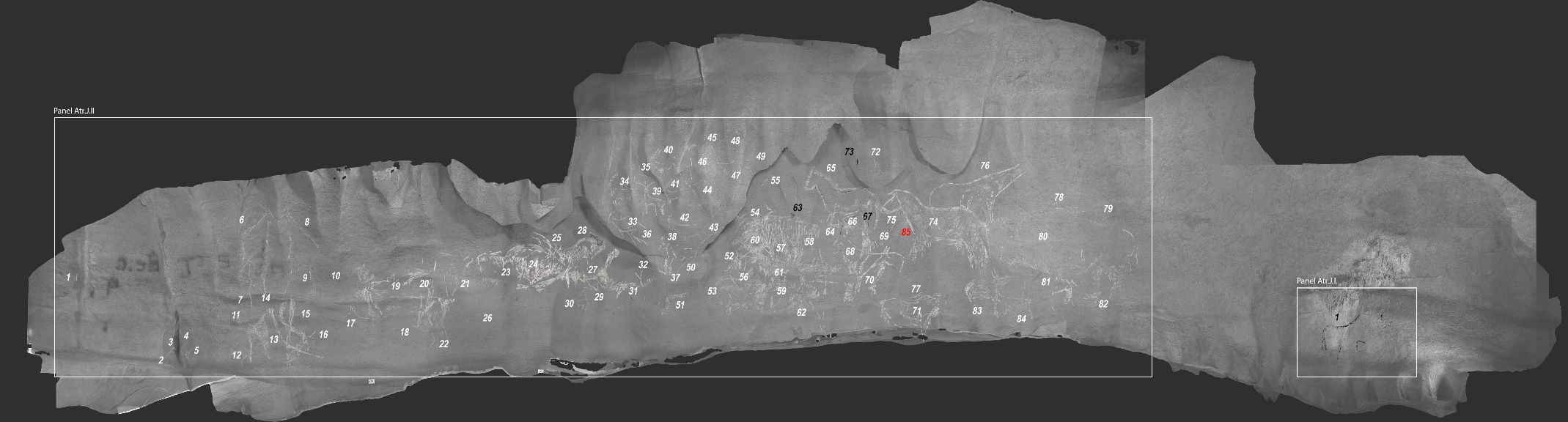
**

***Figure 1, S2* – *Location of the GUs of Sector J****.* * The color of the number indicates the technique: white (engraving or engraving and painting); black (black paint); Red (red paint).

***S3: Rock art stratigraphy.***

The decorated panel of the "Ledge of the Horses" is made up of eighty-four graphic units of which forty-seven are animal representations and the rest are non-figurative representations. Although they are distributed along 9.68 meters in length, direct contact has been detected in 49 of them. All contact points have been analyzed by microscopic observation (*Dinolite^©^ AM7915MZTLEDGE*) and microphotogrammetry (*Agisoft^©^ Metashape Pro*) to determine the order of execution between the graphic units. In this way, we have dumped the parietal stratigraphic information on a Harris matrix (*Harris Matrix Composer^©^*) that allows us to sequence the temporal order of panel decoration for the stratified graphic entities, using *Adobe^©^ Photoshop CC* for the graphic output.


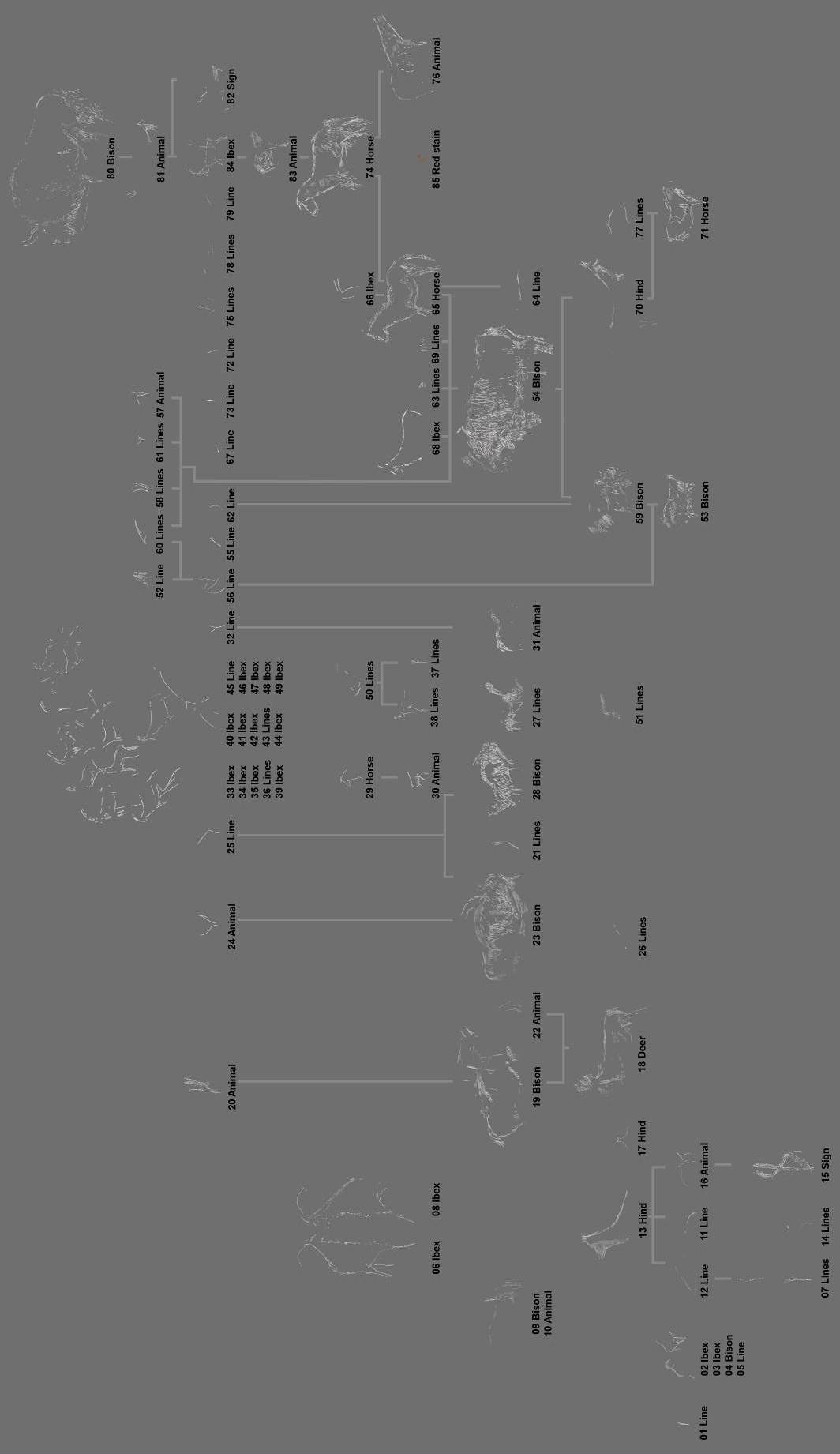


***Figure 1, S3 – Matrix Harris of the J.II decorated panel.***

***S4: Rock art analyses.***

1. **TYPOMETRIC ANALYSIS OF THE ENGRAVED GROOVES**

Archaeological and experimental traces were observed with a *Dino-Lite AD-7013MZT* series digital optical microscope (10x, 250x). For the acquisition of three-dimensional data, we used near-objective photogrammetry with a *Nikon D7100* camera integrating an *AF-S DX Micro NIKKOR 40mm f/2.8G* macro lens incorporating external flashes flush to the support (*Nikon Speedlight SB-910*). The photogrammetric survey and the extraction of different profiles of each trace for subsequent analysis were performed with *Agisoft^©^ Metashape Pro*. In order to analyse the morphology of the incisions from the point of view of the traceology of the incision, the section of the trace was obtained from the Digital Elevation Model (DEM) generated by the micro-photogrammetric model (Ruiz et al. 2019)*.*

1. **EXPERIMENTAL COMPARISON PROGRAM OF THE ENGRAVED GROOVES**

The experimental set was formed by twelve pieces, including burins, bladelets, and broken blades produced by J. A. Munúa using Flysch and Bergeracois flint. The experiment was made by O. Rivero, P. García-Bustos, J. Rios-Garaizar y D. Garate. A right-handed engraver made 10 vertical single tracings, then another right-handed made series of fifty and one hundred tracings in aback-and-forth movement with the same tools. Seven of the ten single tracings were made with an unused active part of the tool, and three of them after the multiple series. This aimed to find differences in tracing execution between unused and worn tools. The profiles of the experimental grooves have been analysed with the same methodology applied for archaeological ones.

1. **SPATIAL ANALYSES OF ROCK ART**

We have performed some visibility analysis using Geographical Information Systems (GIS) and the illuminance value from our experimental torches based on the archaeological remains of Atxurra Cave (Medina-Alcaide *et al*., 2021). We have used the software *ArcGIS’^®^ ArcMap^©^* and *ArcScene^©^*. Firstly, the viewshed of each graphic unit (GU) was analysed, confirming that the figures with the highest visibility in the cave are those found in the “Ledge of the Horses” (sector J) (Intxaurbe *et al*., 2020). In addition, a study of Lines of Sight (LOS) and capacity was carried out (Intxaurbe *et al*., 2022), based on previous experiences (Pastoors and Weniger, 2011; Landeschi *et al*., 2016). And finally, we carried out an accessibility analysis, using a method expressly designed to determine the difficulty of access to cave art, to know the optimal transit routes inside the cave and to estimate the employed time to reach a sector (Intxaurbe *et al*., 2021).

Regarding the capacity, this was analysed employing the estimation of the architect E. Neufert (1951), who indicates that a person standing occupies an area of 0.77 m2 and 1.75 lying down. Ruiz-Redondo (2014) proposed an additional measurement of 0.90 m2 square meters for people sitting or squatting. Prior to this, we defined the sector as the space from which any of the figures would be visible, taking into account the lighting systems employed in the Prehistory.

***S5: Survey and excavation techniques.***

First, we started with a photogrammetric survey of the surface. The photogrammetric equipment used included a *Sony Alpha 700* and a *Sony A7* (along with different calibrated lenses), a *GIGAPAN EpicPRO* mount, and various light sources with a Colour Rendering Index (C.R.I.) of 95% or above. The exposure control was carried out after a radiometric exposure of at least 12 bits per strip, and Profile Connection Space (PCS), Color Management Module, and *Adobe^®^ CMM* were used for colourimetric management.

The surface to be excavated was marked out, leaving a margin of about 10-15 cm with respect to the wall of the engravings. An artificial mesh was established starting from a zero point that is virtually inside the karst massif. A topography base was established a few meters inside the gallery (ATX.101 X: 100.000 Y: 100.00 Z: 100.00). The photogrammetric targets were coordinated using a topographic total station (model *Leica^®^ TC307*) in order to proceed to the modelling of the excavation surface. An exhaustive survey of the surface of the ledge was carried out prior to the excavation, with the objective of locating and surveying with coordinates all the surface remains.

The excavation process was carried out progressively, from the S of the ledge towards the north. A grid system was not followed due to the particularly elongated and irregular morphology of the ledge. The system followed was similar to the one used in Aranbaltza II (see Rios-Garaizar *et al*., 2022). All the localized materials were collected with coordinates, and the elongated materials were recovered with two coordinates, one for each end of it. Record numbers were assigned correlatively, changing series numbers each time a new detritic layer was reached. The charcoals collected manually were at least 0.5 centimetres in size while the smaller fragments were recovered by water recovery techniques. The sediment from areas of 25 cm of diameter was also recovered with a coordinate and log number and soil was stockpiled for later flotation. Water recovery was made using a 1-millimeter mesh sieve for 4/5 of the samples, and using a 0.25-millimeter sieve to recover smaller elements such as microcharcoals and micromammals. Afterwards, the sediment was dried, shielding it from direct exposure to sunlight, and the recovered archaeological material was then sorted.

The Ledge is divided into three zones. The first zone, located to the south/east, is wide and flat, with a slight slope to the east, and is located in a lower position than the next zone. This second zone, which is accessed from the first by a step, is longer and narrower and is in a higher position than the previous one. On this surface the entire N side is elevated above the rest, forming a kind of ridge, which creates a very narrow corridor between the ridge and the wall that is filled with sediments. To the west of this corridor narrows considerably and gives access by means of a ramp to the third area of the ledge, inclined to the N and to the W. Most of the materials have appeared on the first two ledges.

The documented stratigraphy is very simple, from bottom to top the following units can be observed:

- Unit 4: Sterile terrace level with yellowish altered sediment and alteration by manganese precipitation. It contains centimetric boulders of sandstone and limonite and fills the paleotopography of the limestone floor of the ledge.
- Unit 3: Sterile level with siltstone gravels and reddish-brown sandy-loamy matrix. The level has a net contact with Unit 2 and overlies the boulders of the underlying terrace.
- Unit 2: Thin layer 1-1.5 cm thick, formed by light brown decalcification clays and silts. The roof is somewhat hardened and represents a paleosurface on which archaeological materials are deposited. This paleosurface presents a polygonal grid of desiccation, with cracks filled with carbonate. Towards the base, this sediment is slightly carbonated and has a more yellowish hue.
- Unit 1: Current soil, about 2-3 mm thick, with precipitation of maganeses and guano, forming a slightly carbonate surface that at some points is somewhat trampled. The materials of Unit 2 are covered by this very thin layer.

**
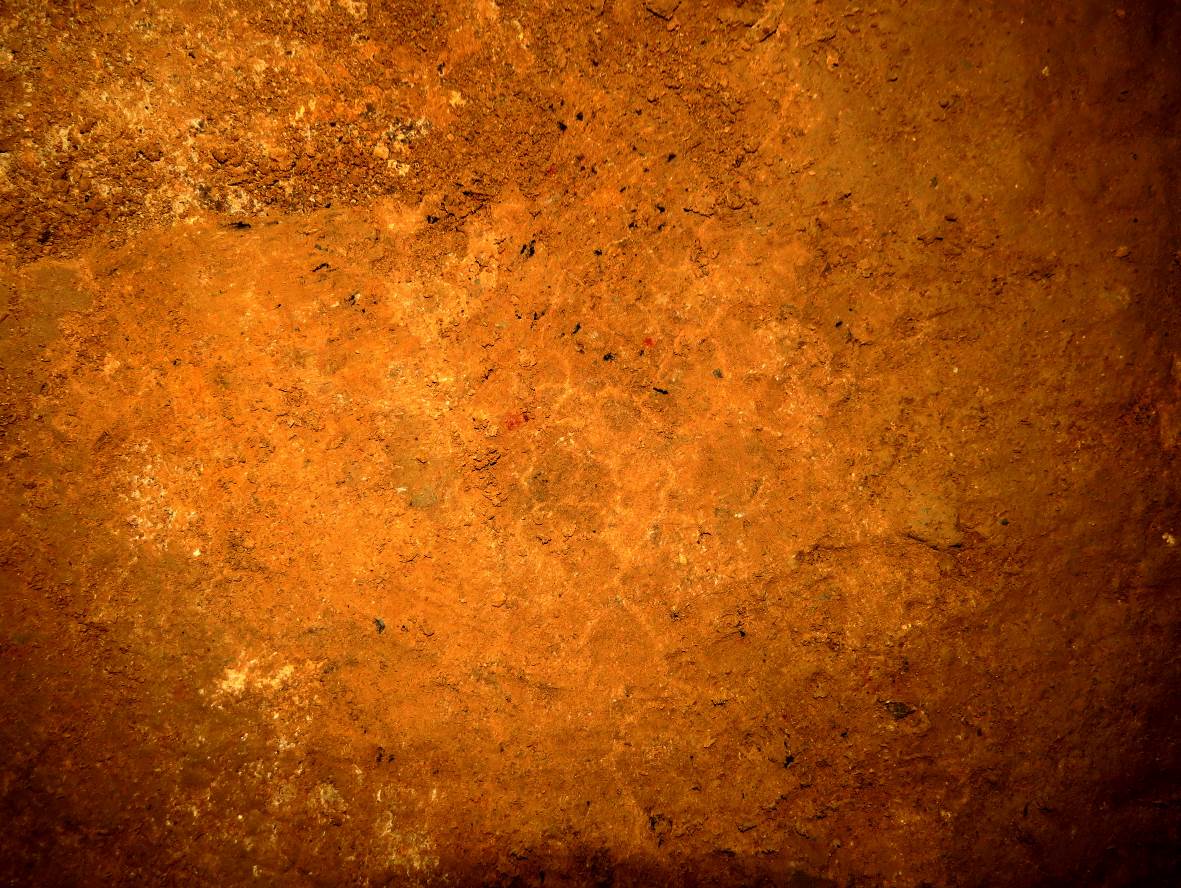
**

***Figure 1, S5 – Roof surface of level 2, with polygonal grid of desiccation, the cracks are filled with carbonate.***


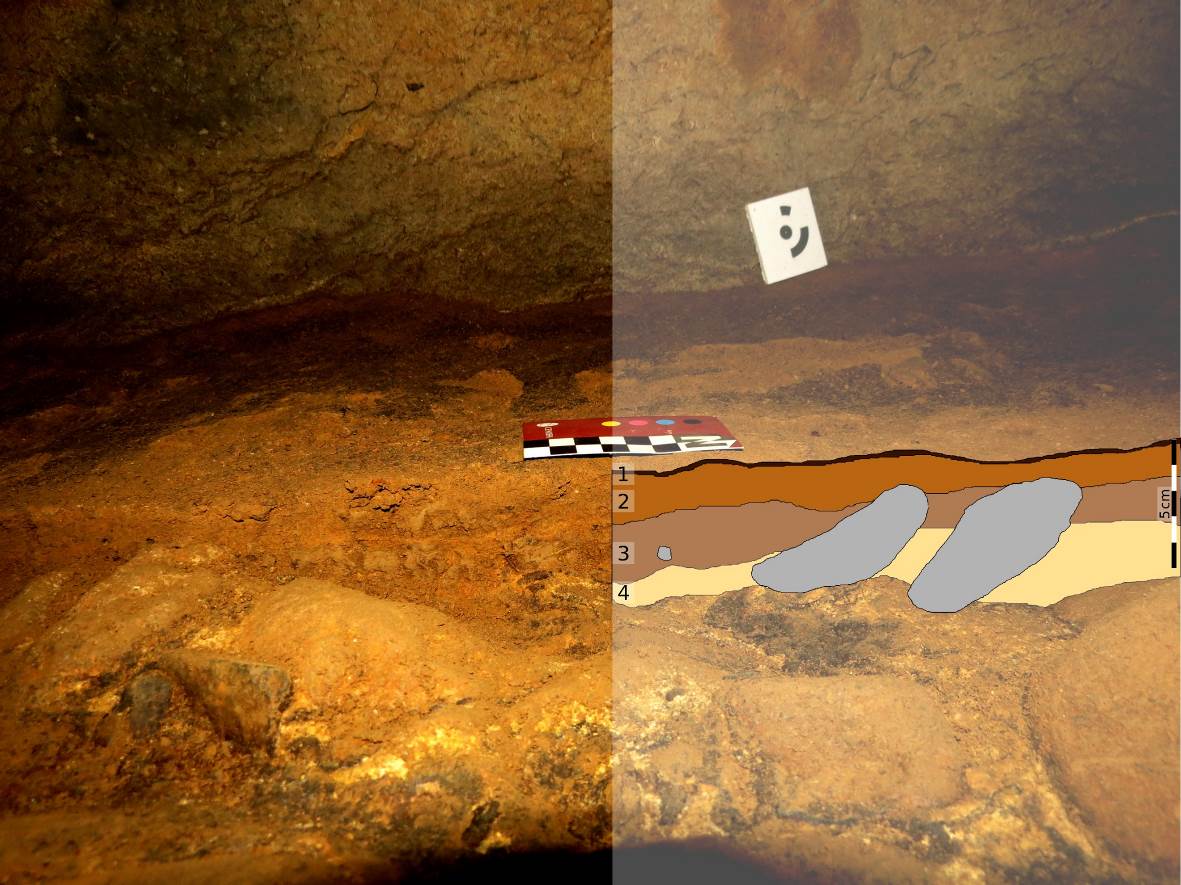


***Figure 2, S5 – Stratigraphic section of the Ledge deposit.***

***S6: Lithic analyses.***

1. **MATERIALS AND METHODS**

Archaeological lithic assemblage is composed by twenty-two remains (Table 1, S6). It has been analyzed following the Integral Lithic Analysis perspective (Rios-Garaizar, 2007) to understand technological organization, including the selection and integration of different strategies of production, use and transport (Nelson, 1991). Raw material provenance was determined according to the flint-types defined by Tarriño-Vinagre (2006) and Rissetto (2009) and by comparing the archaeological samples with geological samples stored at LITHO (CENIEH’s Lithotheque), and the systematics defined for other Upper Palaeolithic assemblages (Rios-Garaizar et al., 2011, 2015, 2019a, 2020a, 2020b, San Emeterio and Rios-Garaizar, 2017).

| **Support type** | Flysch flint | Non determinable flint | North pyrenean flint | Traslucent flint | Lydite | **Total** |
| --- | --- | --- | --- | --- | --- | --- |
| Blade | 4 |  | 1 |  |  | **5** |
| Bladelet | 1 |  |  |  | 1 | **2** |
| Burin spall | 1 |  |  |  |  | **1** |
| Chunk |  |  | 1 |  |  | **1** |
| Chips | 6 | 5 | 1 | 1 |  | **13** |
| **Total** | **12** | **5** | **3** | **1** | **1** | **22** |

***Table 1, S6 – Technological composition of the lithic assemblage.***

The use-wear analysis of the archaeological and experimental pieces was carried out in the Prehistoric Technology laboratory of CENIEH, combining inspection at low magnification (0.7-11.5×) using an *Olympus SZ×16* binocular, and at high magnification (50× -400×) using an *Olympus BX51* metallographic microscope. Microscopic pictures were obtained using a *Nikon camera (DS-8Fi2)* and *NIS-Elements D 4.13.04* software. The obtained multifocal images were reconstructed using *Helicon Focus 6* software. The description of the traces and the identification of the polishes were performed following standardized procedures (González Urquijo and Ibañez Estévez, 1994, Ortega et al., 2006; Rios-Garaizar, 2008, 2010, 2012; Rios-Garaizar et al., 2019b; Rios-Garaizar and Ortega, 2014). The observation and description of the traces generated in the experimental materials has allowed us to obtain a specific reference collection of different types of engravings in soft limestone. The repeated use of the pieces has allowed a good development of the traces, although in some cases hard stone engraving traces have been generated when the engravings exceed the soft outer layer of the limestone and touch the harder inner layers. In addition to the comparison with the experimental pieces, other published experimental collections (González Urquijo and Ibañez Estévez, 1994, Marreiros et al., 2015 and references therein) and the experimental collection (CET) of CENIEH have been used as reference.

1. **RESULTS**

From a technological point of view, although the assemblage is small, we can point out several interesting issues. In the first place, all the recovered remains, except one of lidite, are made on flint, emphasizing the Flysch flint (thirteen remains), as opposed to exogenous varieties (four remains) which include a blade with inverse retouch and the distal fragment of the bec, which have been made on a variety of flint infrequent in the Upper Paleolithic record of the region, whose origin we cannot specify with exactitude. The other remains are patinated and it has not been possible to establish their origin. The recovered blades are regular, with four particularly wide specimens (between 19 and 28 mm). These wide blades have been identified in the Upper Magdalenian from other sites in the area, such as the cave entrance occupation of Atxurra, Armiña or Abittaga, although in none of these sites we have been able to identify the cores from which these blades were produced. The knapping technique is direct percussion with a soft hammer and possibly with a precise preparation of the cornices by abrasion. The transformation by retouch is very slight, in such a way that, except for the dihedral angle burin and the Dufour blade, the recovered tools present slight retouches that barely modify the pieces, accommodating gripping zones and slightly configuring active zones. The splints and small fragments recovered must be put in relation to the configuration of the tools and their use. It is interesting to note how, despite having excavated almost the entire deposit and its good preservation, there are chips and fragments that do not match the supports and tools recovered, suggesting that part of the tools used and configured on the Ledge were taken from there. Likewise, we have not recovered any burin strikes that clearly trace back to the recovered burin, although we have indications that it was revived before a last use that was not very intense.

The experimental wear-traces are very distinctive. Those that have more contact with the hard limestone (Figure 1, S6A and B) develop a bright and flat polish, with deep striations that creates a sort of “tiled” distribution. These traces are concentrated along the active zone and are not very invasive. The other kind of polish was generated in contact with soft limestone making wider grooves. This developed a rough polish, quite bright and with less deep, albeit abundant, striations (Figure 1, S6D - F). There are also pieces which develop a rough polish with some flat and brighter areas, which can be interpreted as an intermediate situation between the two types of traces experimentally identified (Figure 1, S6C).

***
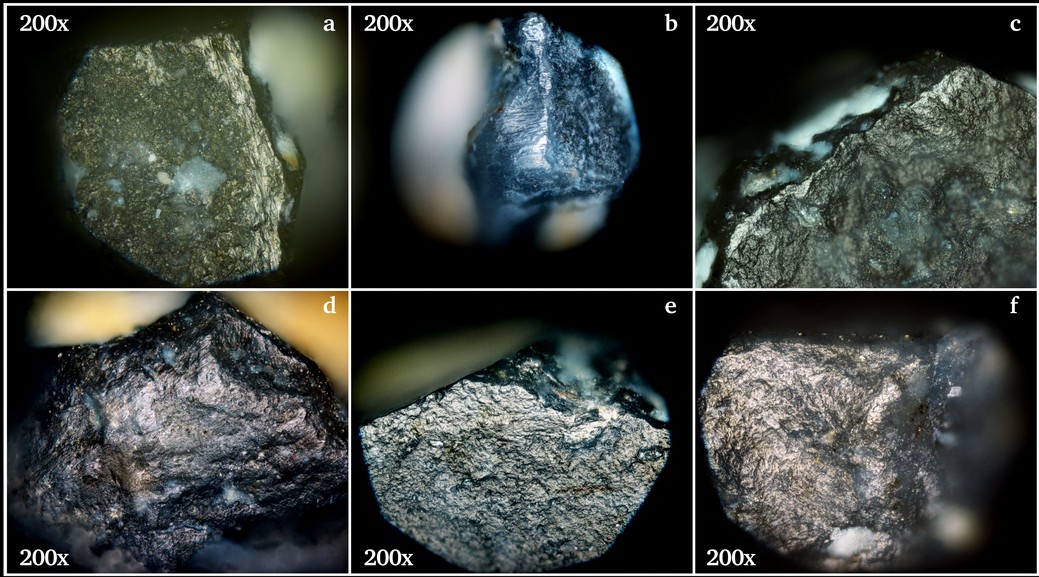
Figure 1, S6 – Experimental traces generated by limestone engraving.***

The functional analysis revealed traces of use only in the five laminar supports. The conservation of the traces is good, and only some chemical alteration of the surfaces due to atmospheric exposition has been observed. The traces are located on the distal ends of pointed pieces (two cases), on the trihedrons formed by fractures (two cases), on a burin dihedral (one case) and on the sides of the blades (3 cases) (Table 2, S6). In all cases the traces are related to the work on a medium hard and abrasive material, being traces of scraping, engraving and incision. The comparison with the experimental traces obtained in the soft limestone engraving suggests that all these pieces were used in the preparation and execution of the engraved figures. Thus, we have seen traces in lateral scraping edges, with wide extensions, which suggest preparatory scraping of the wall; incision traces with the fine trihedrons of the fractures, which we can relate to the deep fine engravings with V profile; and traces made with dihedrals or wider trihedrons that possibly have to be related to the wide engravings with U profile and barcode background that compose the main figures of the panel.

| **NR** | **Length (mm)** | **Width (mm)** | **Thickness (mm)** | **Weight (g)** | **Raw material** | **Type** | **Active edge** | **Use** | **Incission section** |
| --- | --- | --- | --- | --- | --- | --- | --- | --- | --- |
| ATR.J.1038 | 62 | 23 | 8 | 12.83 | Flysch Flint | Dihedral burin on blade | Burin dihedre | Engraving (poorly developed) | Flat |
|  |  |  |  |  |  |  | Left edge | Soft stone scraping | - |
| ATR.J.1034 | 59 | 19 | 7 | 8.26 | Flysch Flint | Broken blade | Pointed end | Soft stone engraving | Flat |
|  |  |  |  |  |  |  | Botom left dihedre (fracture) | Soft stone engraving | V-shaped |
|  |  |  |  |  |  |  | Botom rigth dihedre (fracture) | Soft stone engraving | V-shaped |
| ATR.J.1132 | 56 | 22 | 7 | 9.25 | Exogenous Flint | Broken blade with partial ventral retouch | Pointed end | Soft stone engraving | Flat |
|  |  |  |  |  |  |  | Left edge | Soft stone scraping | - |
|  |  |  |  |  |  |  | Right edge | Soft stone scraping | - |
| ATR.J.1120 | 72 | 14 | 6 | 5.36 | Flysch Flint | Blade | Pointed end | Soft stone engraving | Flat |
| ATR.J.1023 | 60 | 28 | 4 | 8.59 | Flysch Flint | Broken blade | Botom left dihedre (fracture) | Soft stone engraving | V-shaped |
|  |  |  |  |  |  |  | Botom rigth dihedre (fracture) | Soft stone engraving | V-shaped |

***Table 2, S6 – Use-wear results***.

Functional analysis of lithic tools recovered in rock art contexts has provided, in some cases, examples of tools used in limestone engraving (Plisson, 2005; López-Tascón et al., 2020), although other uses are also frequently documented (Rios-Garaizar et al., 2015). In the nearby Ekain Cave, traces of engraving have been documented on a burin recovered from a ledge of the cave (Altuna pers. comm.) and in the Alkerdi II Cave, the first results obtained have identified traces that may be related to the process of graphic activity on the walls of the cavern (personal obs.). In Armiña site, located in the same karstic system than Atxurra, we have recognized an occupation in an internal context that we associate with activities of a symbolic nature (Rios-Garaizar et al., 2020b). Among the materials recovered by J.M. Barandiarán in 1926 that is labelled as coming from the Armiña Cave are two retouched flint blades with traces compatible with limestone engraving very similar to those from the “Horses’ Ledge” (Rios-Garaizar et al., 2020b).

**
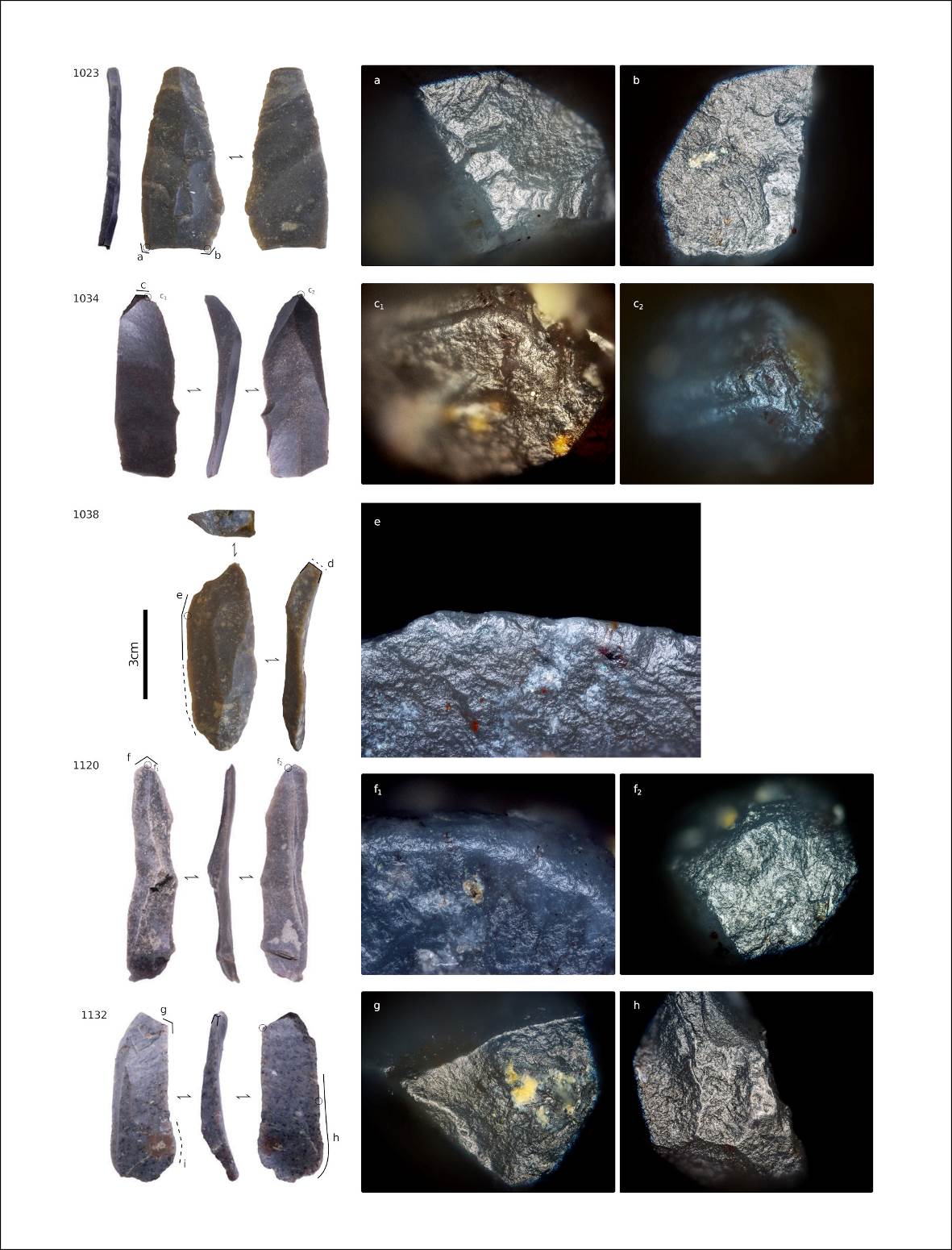
**

***Figure 2, S6 – Use-wear traces on the archaeological materials.***

***S7: Charcoal analyses.***

1. **ANTHRACOLOGY**

Anthracological analyses were conducted to determine the taxonomy of the wood charcoal (Chabal *et al.*, 1999). To identify the anatomical and biometric properties of the wood specimens, clean cuts were made on each fragment to obtain transversal, tangential and radial sections. The classification of each specimen and the alterations were determined by comparison with various reference sources and papers (Schweingruber, 1990; Vernet *et al.*, 2001). A *BA410* Motic light microscope was used with light reflected to a light-dark reflection field at magnifications ranging from 40 to 600x. To carry out a more optimal observation of the sample, at higher magnifications we used a scanning electron microscope (S.E.M). We undertook this activity mostly at the Central Service for Research Support (SCAI) at the University of Cordoba (UCO), where we used a model *JEOL JSM 6300*, after double gold-plating the charcoal samples so that they can conduct better inside the microscope. The samples were placed in several cylindrical SEM sample carriers and were affixed to an electrically conductive resin (Temfix Adhesive-type) to hold them in place, so that we could observe the desired section. Several studies have pointed out the importance of anthracological analysis prior to 14C-AMS dating, in order to determine chrono-ecological coherence and detect possible taphonomic problems (for example, Carrión *et al.*, 2018). All charcoal samples selected to 14C-AMS dating were anthracologically analysed in a light microscope before sending them to the laboratories.

1. **14C-AMS DATING**

7 wood charcoal fragments have been analysed by Beta Analytic Laboratory for 14C-AMS dating and 1 wood charcoal in University of Oxford (ORAU). These laboratories used the ABA (acid-base-acid) pre-treatment protocol (Brock *et al.* 2010, http://www.c14dating.com/pret2.html). We used *Oxcal 4.4*^©^ online software, firstly, to calibrate the radiocarbon dates in calendar years using an IntCal20 calibration curve (Reimer *et al.* 2020). Next, this program allowed us to evaluate through Bayesian statistics the different phases of human occupation in the cave, both for the exterior chambers and the interior spaces (Ledge of Horse and Armiña site). In particular, we employed the Charcoal Outlier model in order to characterise the existence of the atypical values following Bronk *et al.* 2009.

|  | **LEDGE OF THE HORSES** | | | | | |  |
| --- | --- | --- | --- | --- | --- | --- | --- |
| **Number** | **REFERENCE** | **DATING TYPE** | **CONVEN. AGE** | **INTCAL 20 (CALBP 95,4%)** | **DELTA C13** | **NATURE** | **REFERENCE** |
|  | ***Scattered charcoals (torches)*** | | | | | | |
| 1 | Beta-503388 | AMS-Standard delivery | 8,620±30 | 9,677-9,532 | -25,60 | Wood Charcoal (*Deciduous Quercus*) | Unpublished |
| 2 | OxA-36937/  P-443(ORAU) | AMS-Standard delivery | 8,876±37 | 10,179-9,784 | -27,97 | Wood Charcoal (*Deciduous Quercus*) | Unpublished |
| 3 | Beta-503387 | AMS-Standard delivery | 8,920±40 | 10,192-9,905 | -25,80 | Wood Charcoal (*Salix* sp.) | Unpublished |
| 4 | Beta-522187 | AMS-Micro-sample Analysis; Standard delivery | 9,040±50 | 10,334-9961 | NA | Wood Charcoal | Unpublished |
| 5 | Beta-523313 | AMS-Micro-sample Analysis; Standard delivery | 12,380±40 | 14,840-14,190 | -22,60 | Wood Charcoal (*Juniperus* sp.) | Unpublished |
|  | ***Fireplace 2*** | | | | | | |
| 6 | Beta-503386 | AMS-Micro-sample Analysis; Standard delivery | 11,690±50 | 13,740-13,449 | NA | Wood Charcoal (*Juniperus* sp.) | Unpublished |
| 7 | Beta-522186 | AMS-Micro-sample Analysis; Standard delivery | 12,330±60 | 14,832-14,086 | -23,00 | Wood Charcoal | Unpublished |

|  | **EXTERNAL SITE** | | | | | | |
| --- | --- | --- | --- | --- | --- | --- | --- |
| **Number** | **REFERENCE** | **DATING TYPE** | **CONVEN. AGE** | **INTCAL 20**  **(CALBP 95,4%)** | **DELTA C13** | **NATURE** | **REFERENCE** |
| 8 | Beta-425446 | AMS-Standard delivery | 12,420±40 | 14,885-14,276 | -20.6 o/oo | Bone | Unpublished |
| 9 | Beta-404444 | AMS-Standard delivery | 12,450±40 | 14,940-14,316 | -20.7 o/oo | Bone | Unpublished |
| 10 | Beta-404443 | AMS-Standard delivery | 12,550±40 | 15,112-14,548 | -20.7 o/oo | Bone | Unpublished |
| 11 | Beta-456229 | AMS-Standard delivery | 13070±40 |  | ¿ | Bone | Unpublished |

|  | **ARMIÑA ENTRANCE** | | | | | | |
| --- | --- | --- | --- | --- | --- | --- | --- |
| **Number** | **REFERENCE** | **DATING TYPE** | **CONVEN. AGE** | **INTCAL 20**  **(CALBP 95,4%)** | **DELTA C13** | **NATURE** | **REFERENCE** |
| 12 | Beta-456230 | AMS-Standard delivery | 12,300±40 | 14,840 -14,190 | -25,60 | Bone | Rios-Garaizar *et al.* 2020 |
| 13 | Beta-498136 | AMS-Standard delivery | 12,390±30 | 14,846-14,209 | -27,97 | Bone | Rios-Garaizar *et al.* 2020 |

***Table 1, S7 – 14C-AMS data included in the Bayesian analysis calibre with Oxcal 4.4. online, using IntCal20 dataset.***

- **Plot:**

Plot()

{

Outlier_Model("Bone", T(5), U(0,4),"t");

Outlier_Model("Charcoal",Exp(1,-10,0),U(0,3),"t");

Sequence("Atxurra-Armiña")

{

Boundary("Start Phase 1")

{

color="blue";

};

Phase("1")

{

R_Date("Beta-456229", 13070, 40)

{

Outlier("Bone",0.05);

};

R_Date("Beta-404443", 12550, 40)

{

Outlier("Bone",0.05);

};

R_Date("Beta-404444", 12450, 40)

{

Outlier("Bone",0.05);

};

R_Date("Beta-425446", 12420, 40)

{

Outlier("Charcoal",1);

};

R_Date("Beta-498136", 12390, 40)

{

Outlier("Charcoal",1);

};

R_Date("Beta-456230", 12300, 40)

{

Outlier("Bone",1);

};

R_Date("Beta-523313", 12380, 40)

{

Outlier("Bone",1);

};

R_Date("Beta-522186", 12330, 60)

{

Outlier("Charcoal",1);

};

R_Date("Beta-503386", 11690, 50)

{

Outlier("Charcoal",1);

};

};

Boundary("End Phase 1")

{

color="blue";

};

Interval("Transition Phase 1/2");

Boundary("Start Phase 2")

{

color="blue";

};

Phase("2")

{

R_Date("Beta-522187", 9040, 50)

{

Outlier("Charcoal",1);

};

R_Date("Beta-503387", 8920, 40)

{

Outlier("Charcoal",1);

};

R_Date("OxA-36937", 8876, 37)

{

Outlier("Charcoal",1);

};

R_Date("Beta-503388", 8620, 30)

{

Outlier("Charcoal",1);

};

};

Boundary("End Phase 2")

{

color="blue";

};

};

};

***Table 2, S7 – Bayesian analysis: Two phases: “high agreement”.***

**
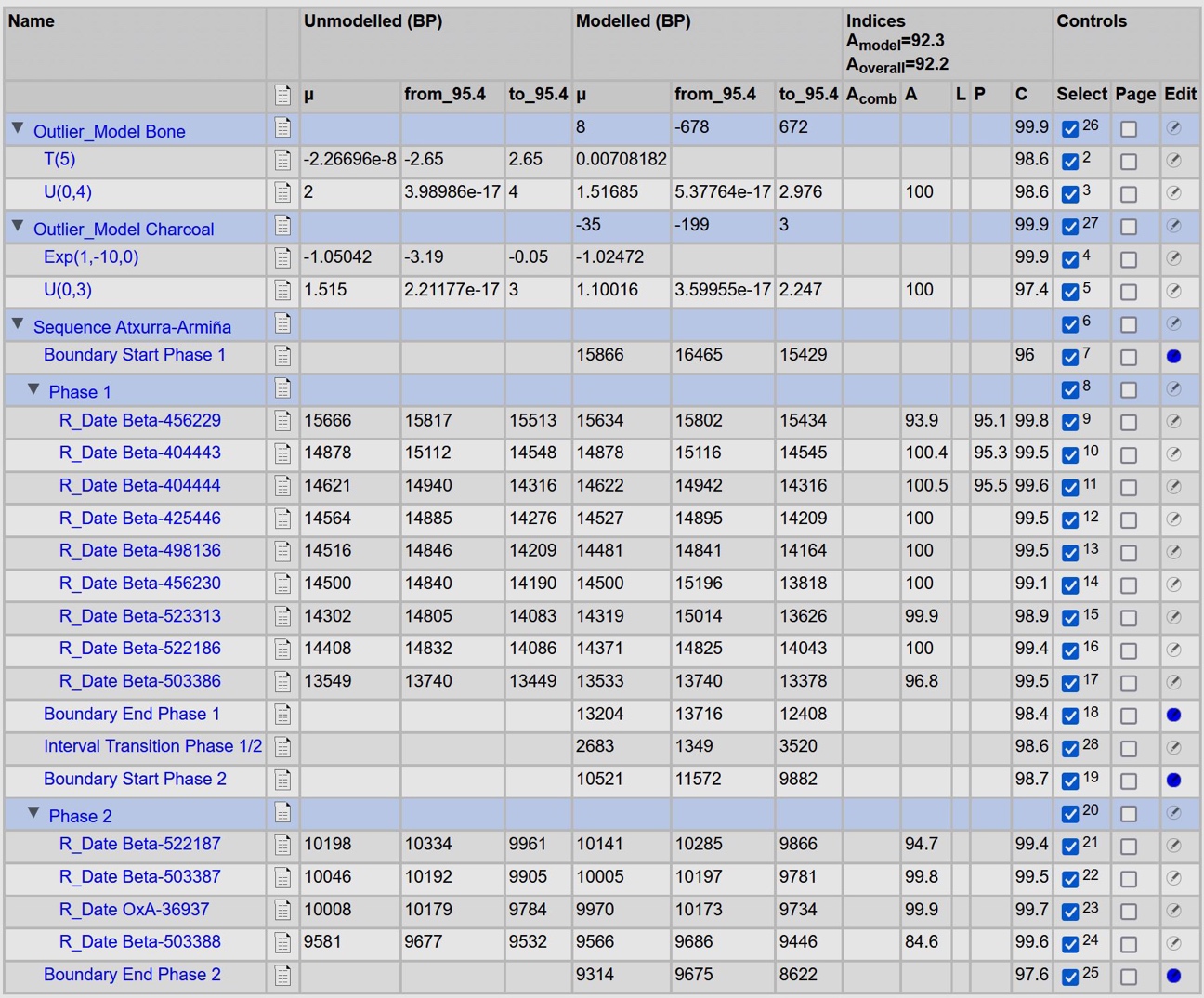
**

**Plot**

Plot()

{

Outlier_Model("Bone", T(5), U(0,4),"t");

Outlier_Model("Charcoal",Exp(1,-10,0),U(0,3),"t");

Sequence("Atxurra-Armiña")

{

Boundary("Start Phase 1")

{

color="blue";

};

Phase("1")

{

R_Date("Beta-456229", 13070, 40)

{

Outlier("Bone",0.05);

};

R_Date("Beta-404443", 12550, 40)

{

Outlier("Bone",0.05);

};

R_Date("Beta-404444", 12450, 40)

{

Outlier("Bone",0.05);

};

R_Date("Beta-425446", 12420, 40)

{

Outlier("Charcoal",1);

};

R_Date("Beta-498136", 12390, 40)

{

Outlier("Charcoal",1);

};

R_Date("Beta-456230", 12300, 40)

{

Outlier("Bone",1);

};

R_Date("Beta-523313", 12380, 40)

{

Outlier("Bone",1);

};

R_Date("Beta-522186", 12330, 60)

{

Outlier("Charcoal",1);

};

};

Boundary("End Phase 1")

{

color="blue";

};

Interval("Transition Phase 1/2");

Boundary("Start Phase 2")

{

color="blue";

};

Phase("2")

{

R_Date("Beta-503386", 11690, 50)

{

Outlier("Charcoal",1);

};

};

Boundary("End Phase 2")

{

color="blue";

};

Interval("Transition Phase 2/3");

Boundary("Start Phase 3")

{

color="blue";

};

Phase("3")

{

R_Date("Beta-522187", 9040, 50)

{

Outlier("Charcoal",1);

};

R_Date("Beta-503387", 8920, 40)

{

Outlier("Charcoal",1);

};

R_Date("OxA-36937", 8876, 37)

{

Outlier("Charcoal",1);

};

R_Date("Beta-503388", 8620, 30)

{

Outlier("Charcoal",1);

};

};

Boundary("End Phase 3")

{

color="blue";

};

};

};

**
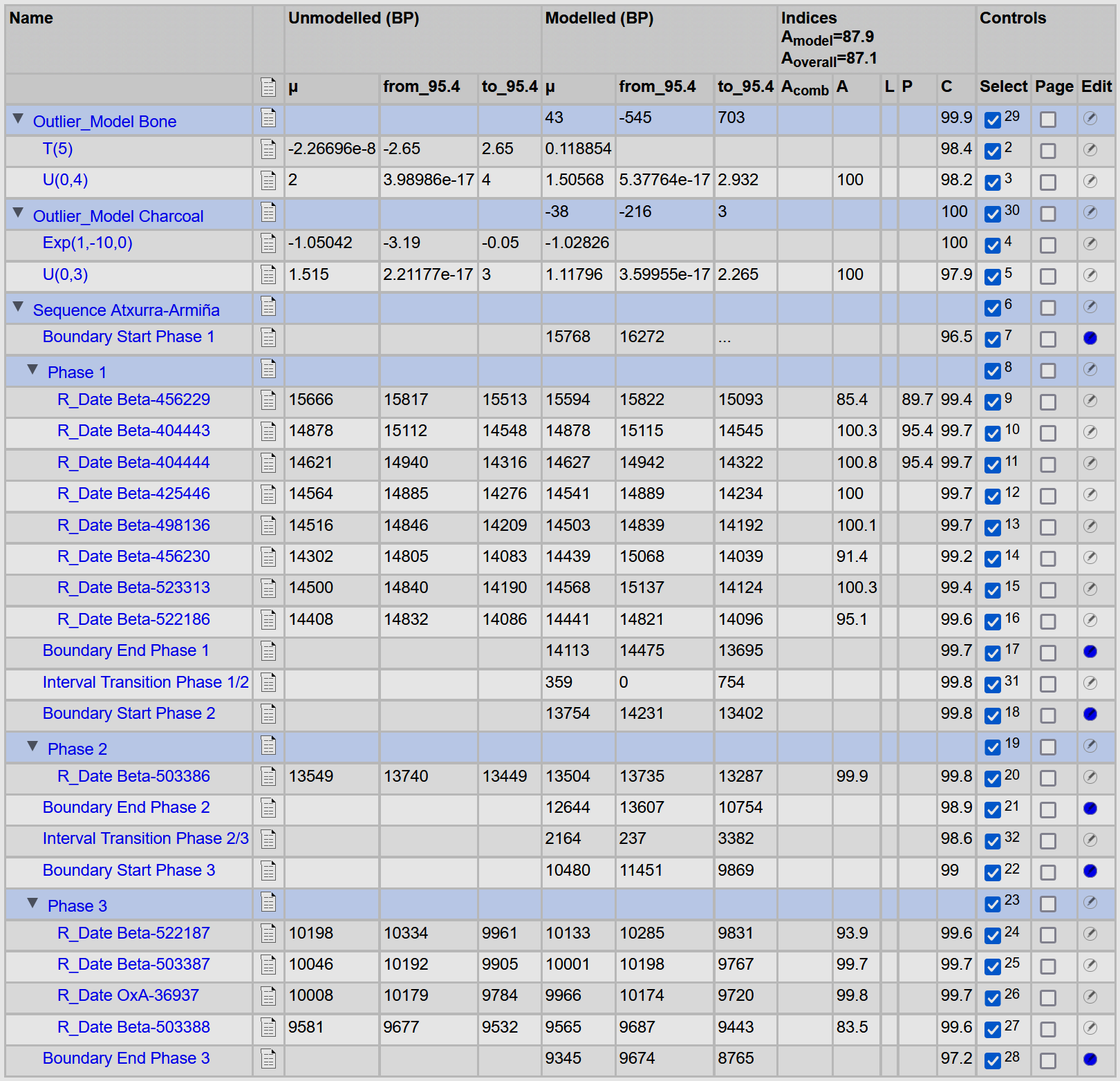
**

***Table 3, S7 – Bayesian analysis: Three phases: "poor agreement".***

***S8: Hearth analyses.***

3 unaltered fireplace samples were taken plastering gypsum from the “Ledge of the Horses”. After drying, they were impregnated by a 7:3 part mixture of polyester resin (*Palatal P4-01*), acetone and 7 mL methylethyl ketone peroxide (MEKP) per litre of polyester resin and acetone mixture, to create three thin sections at the Soil Micromorphology laboratory of CENIEH (Burgos, Spain). The thin sections were studied using an *Olympus BH2* petrographic microscope, equipped with an *Olympus DP10* digital camera and *Nikon Elements* imaging software at the Sedimentary Petrology laboratory of the University of the Basque Country (Leioa, Spain). Thin sections were described according to the terminology used in soil micromorphology (Stoops et al., 2018), sedimentary deposits (van der Meer and Menzies, 2011) and archaeological soils (Nicosia and Stoops, 2017).

| **Rubefaction area** | **Maximum axis (m)** | **Minimum axis (m)** | **Area (**m^2^) | **Perimeter (m)** |
| --- | --- | --- | --- | --- |
| ***Fireplaces*** | | | | |
| **1** | 0,311 | 0,308 | 0,063 | 0,946 |
| **2** | 0,383 | 0,202 | 0,044 | 1,003 |
| **3** | 0,664 | 0,305 | 0,151 | 1,688 |
| ***Torch*** | | | | |
| **4** | 0,030 | 0,001 | 0,00049 | 0,0676 |

***Table 1, S8 – Dimension of fireplaces and torch obtained through ArcGIS.***

**
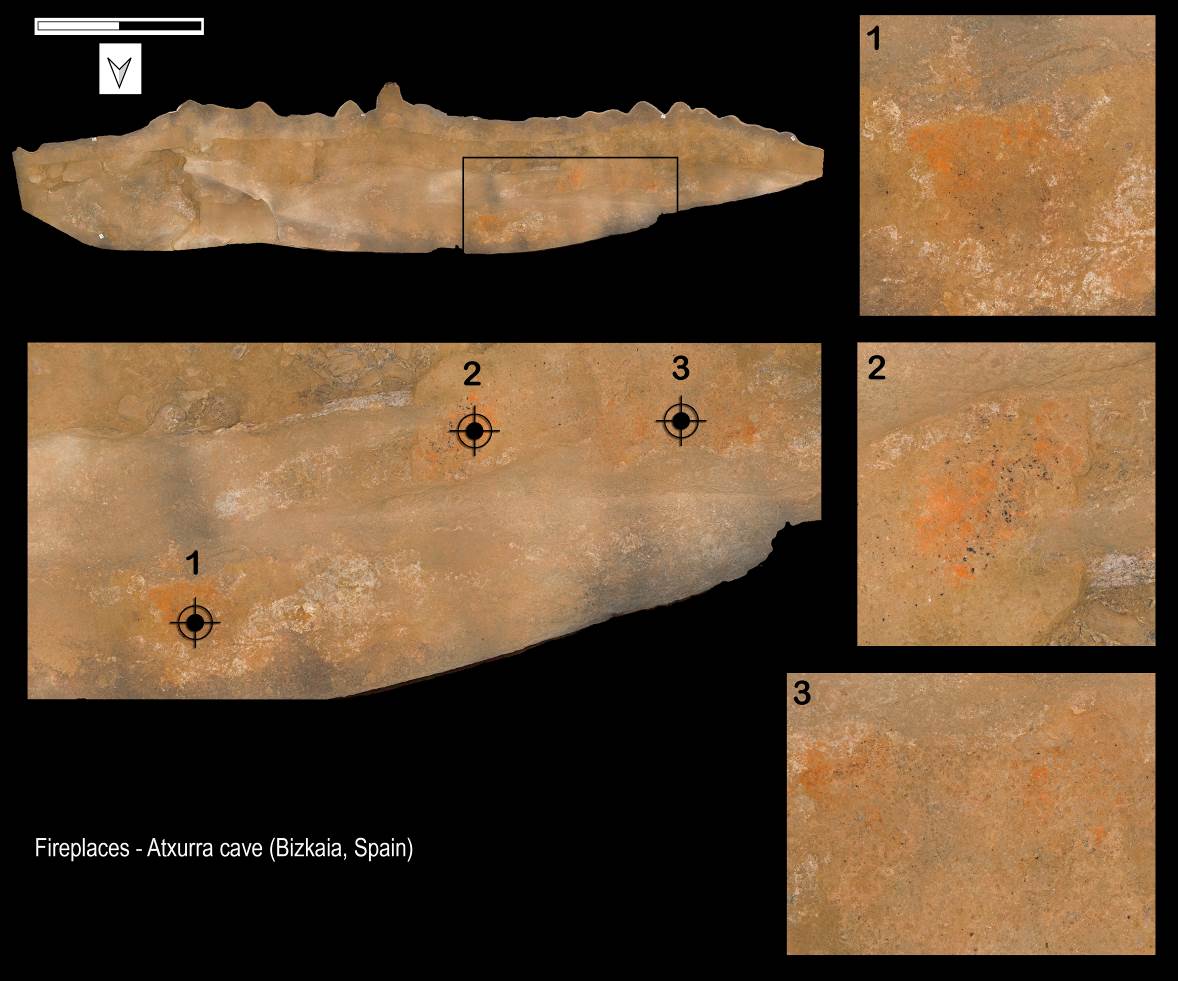
**

***Figure 1, S8 – Fireplaces 1, 2, and 3 (F1, F2 and F3).***

***
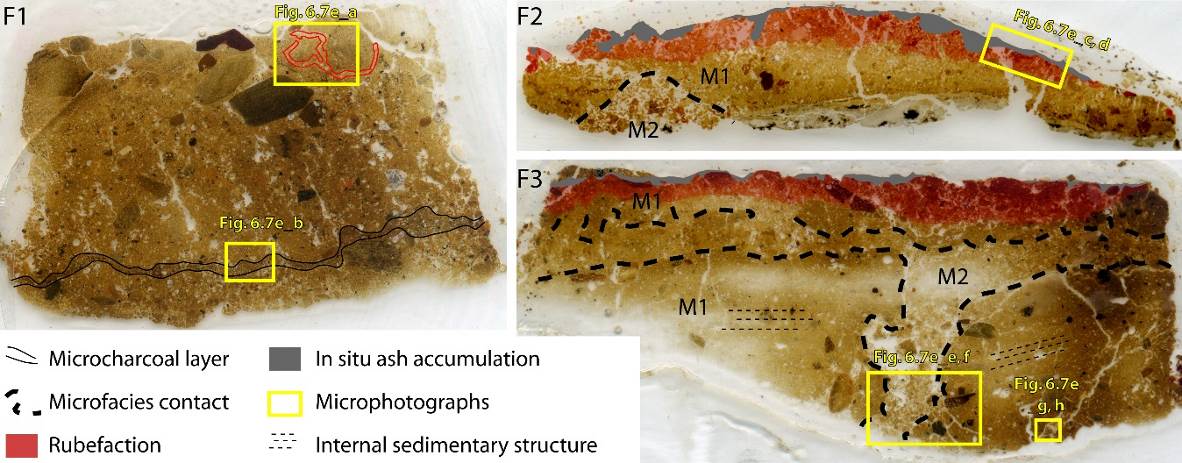
Figure 2, S8 – The three thin section samples (F1, F2 and F3), and their different macro-structures (M1 and M2).***

| **Sample** | **Description** | **Interpretation** |
| --- | --- | --- |
| F1 (H1) | 1 microfacies characterized (M1) by a silt and fine sand matrix-supported fabric. Skeleton grain is mainly compound of partially rounded lutite and sandstone lithoclasts (50%), rip-up clasts (30%) and anorthic ferruginous nodules (20%). Isolated there are also partially burned microfaunal bones, and charcoal (without internal structure), or animal fat at the upper part of the thin section. Either in the upper part of the sample, merged with the groundmass, there are discontinuously arranged 0.4 mm thick ash concentrations (Sup. Fig. 6.7e; a). Additionally, in the lower part of the sample, there is an irregular darkened layer probably due to a microcharcoal concentration (Sup. Fig. 6.7e; b). | The discontinuous ash accumulation into the M1 point out the presence of fire. Because of the absence of rubefaction as well as charcoal or animal fat related to the ash deposits, their origin is most probably related to torches. In this sense, after the falling of the ash from the torches to the floor, those could have been buried in the sediment by footsteps and the sediment carried by them.  The dark irregular layer at the bottom of the sequence could have been formed by the infiltration of microcharcoal through the intergranular porosity by water. |
| F2 (H2) | It is made of 2 microfacies, M1 and M2 (Sup. Fig. 6.7d):  M1 is most abundant and similar to the previous sample microfacie, being silt and fine sand matrix-supported fabric. The top of the thin section, which represents the current floor, is rubefacted, being most of its surface covered by disperse ash remains (Sup. Fig. 6.8e; c, d). Except of some fissure type porosity, there is no other sing of deformation along the rubefacted area.  M2 is localized in the lower area of the thin section (Sup. Fig. 6.7d), being an ash, silt, and fine sand matrix-supported fabric, with an open intergranular porosity and disperse traces of phosphate in the groundmass. Skeleton grain is represented by isolated and altered microfauna bones and rip-up clasts.  The contact between M1 and M2 is regular and clean. | There are two different fire remains and periods. The first one is related to the formation of M2, with the presence of disperse ash and lack of fireplace evidence. We interpret these like ash remains that drop from the torches.  After the formation of M2, comes the formation of M1, characterized by the presence of practically intact rubefaction area with disperse ash remains, in the current floor arrangement, and interpreted as a fireplace. |
| F3 (H3) | It is made of 2 microfacies, M1 and M2 (Sup. Fig. 6.7d):  M1 is similar to F1 microfacie and F2 M1 microfacie, having a silty-fine sand matrix-supported fabric. The upper part of the sequence, current floor, is rubefacted, having most of its surface covered by disperse and concentrated ash remains. Beyond some fissure type porosity, there is not any sing of deformation along the rubefacted area, although a thin and none rubefacted M1 piece is above the in situ ash accumulation. Respect the fissure type porosity along M1, those situated under M2 microfacie are partially filled by ash calcium carbonate crystals (Sup. Fig. 6.7e; e, f, g, h).  M2 is similar to F2 M2 microfacie, having areas with much more phosphate concentration in the groundmass, and other areas enriched in ash calcium carbonate crystals (Sup. Fig. 6.7e; e, f), but all of them represent an open intergranular matrix-supported fabric.  Whether the contact between M1 and M2 is clean in most places, there are sings of post-depositional deformation that make the boundary diffuse in some area, and intruding vertically part of M2 in M1, tilting nearest parts of M1 in the process (Sup. Fig. 6.7d). | Similar to F2, there are at least two different fire remains and periods. The first period is related to M2, which is formed above of M1 with the ash remains drop from the torches, as well as microfaunal bones that are partially burn and phosphatized. By post-depositional compressive processes such as footsteps, occurs fissure type porosity and sediment deformation, whereas the first ones are partially filled by the infiltration of the ash crystals.  After the deformation, a fireplace is made that rubefacted all the surfaces. |

***Table 2, S8 – Micromorphological analysis of each hearth (F1, F2 and F3).***


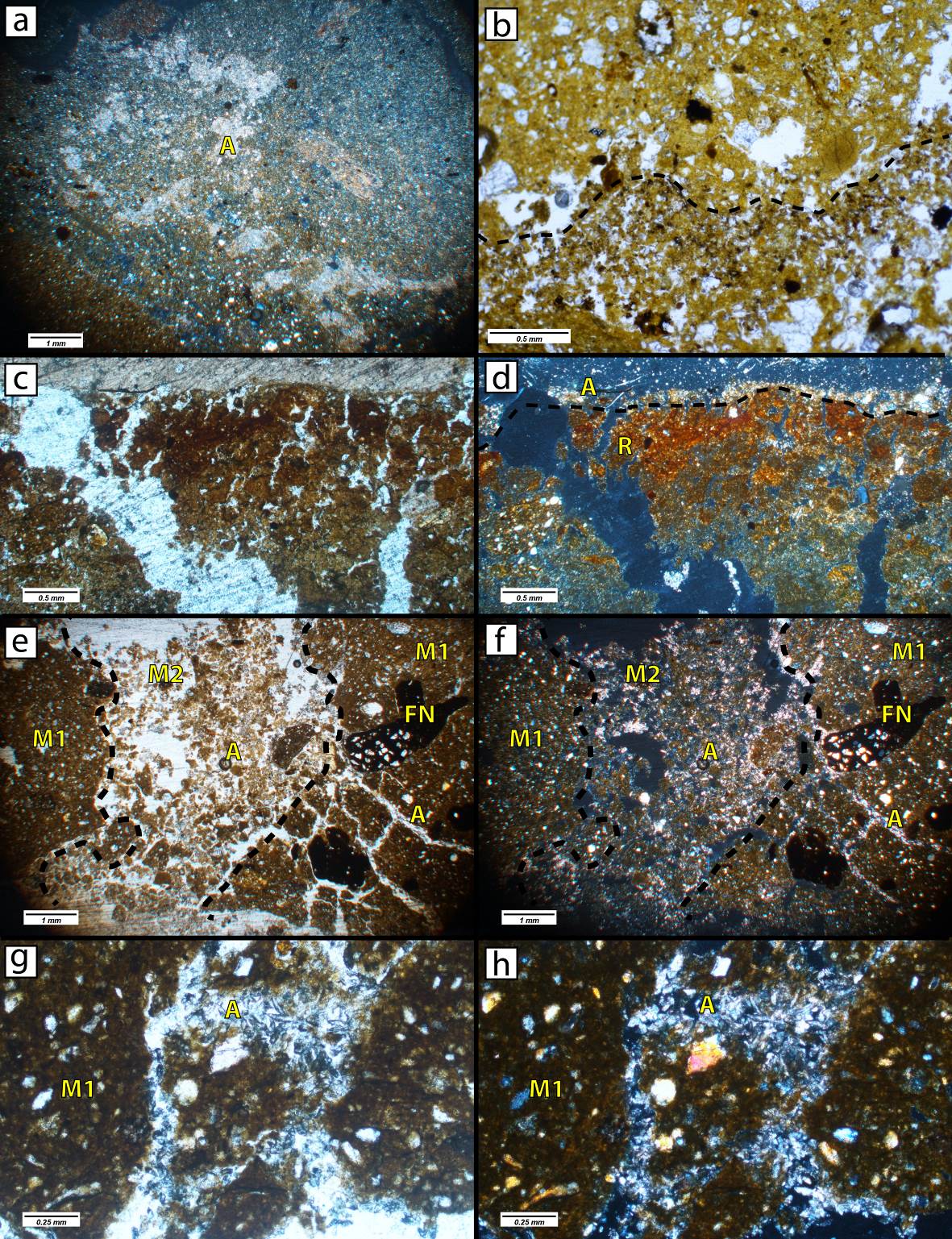


***Figure 3, S8 – Microphotographs of the fireplace thin sections:*** (**A**) Discontinuous concentration of ash into the microfacies, under cross-polarised light (XPL); (**B**) The boundary with the darkened layer; (**C**) In situ fireplace; (**D**) same as (**C**) but under XPL, in where the dispersed ash concentration is detected above the rubefacted area; (**E**) Vertical intrusion of M2 into M1; (**F**) same as (**E**) but under XPL, in where it is possible to distinguish ash crystals filling the fissure type porosity in M1; (**G**) detailed microphotograph of ash crystals partially filling the fissure type porosity; (**H**) same as g) but under XPL. A: ash; R: Rubefacted; FN: Ferruginous nodule; M1: Microfacies 1; M2: Microfacies 2.

***S9: Zooarchaeological analysis.***

1. **MACROFAUNAL ANALYSIS**

In total, the faunal material yielded 69 remains. Two elements were identifiable to taxa (Vulpes vulpes), 14 were identified only to the mammal category, two bird taxa, and 51 were non-identifiable specimens (Tables 1 and 2). The preservation was poor, with bone surfaces significantly altered, limiting the identification of possible taphonomic modifications. The assemblage presents a high state of fragmentation, where 67% of the elements are less than 1cm, 20% are equal to 1cm, and 13% are between 2 and 5cm in length. The faunal composition and its location within the cave could not discard the presence of small carnivores contributing birds into the cave. However, the limited sample and preservation state prevent further identifying the origin of this macrofaunal assemblage.

| **Species** | **NISP** | **%N** |
| --- | --- | --- |
| *Vulpes vulpes* | 2 | 3 |
| Mammal | 14 | 20 |
| Indeterminate | 51 | 74 |
| Bird | 2 | 3 |
| **Total** | **69** | **100** |

***Table 1, S9 – Number of specimens, classified by species category, identified on the Atxurra ledge.***

| **﻿ID** | **x** | **y** | **z** | **Dim. (cm)** | **NISP** | **Specie** | **Element** |
| --- | --- | --- | --- | --- | --- | --- | --- |
| 1032 |  |  |  | 3 | 1 | Bird | Ulna |
| 1036 |  |  |  | <1 | 6 | Unknown | Micro-fragments |
| 1037 |  |  |  | <1 | 1 | Unknown | Teeth? |
| 1049 |  |  |  | <1 | 1 | Unknown | Diaphysis |
| 1057 |  |  |  | <1 | 2 | Unknown | Diaphysis |
| 1057 |  |  |  | <1 | 1 | Unknown | Diaphysis |
| 1061 | 98,45 | 115,45 | 102,09 | <1 | 4 | Unknown | Diaphysis |
| 1071 | 98,71 | 113,94 | 102,02 | <1 | 4 | Unknown | Diaphysis |
| 1094 |  |  |  | 2 | 1 | Mammal | pel/ul/sc |
| 1097 |  |  |  | <1 | 1 | Unknown | Diaphysis |
| 1096 |  |  |  | <1 | 6 | Unknown | Micro-fragments |
| 1099 |  |  |  | <1 | 4 | Unknown | Diaphysis |
| 1103 |  |  |  | 1 | 4 | Mammal | Diaphysis |
| 1103 |  |  |  | 1 | 1 | Unknown | Diaphysis |
| 1103 |  |  |  | 2 | 1 | Bird | Tarsometatarsus |
| 1127 |  |  |  | 1 | 1 | Mammal | Diaphysis |
| 1130 |  |  |  | <1 | 10 | Unknown | Micro-fragments |
| 1130 |  |  |  | 1 | 1 | Mammal | Rib |
| 1151 |  |  |  | 1 | 1 | Mammal | Rib |
| 1152 |  |  |  | <1 | 1 | Unknown | Micro-fragments |
| 1153 |  |  |  | <1 | 3 | Unknown | Diaphysis |
| 1153 |  |  |  | 1 | 2 | Mammal | Diaphysis |
| 1155 | 98,79 | 117,13 | 102,53 | 2 | 1 | Fox | Rib |
| 1156 | 98,7 | 117,42 | 102,48 | 1 | 2 | Mammal | Epiphysis |
| 1157 |  |  |  | 1 | 1 | Mammal | Diaphysis |
| 1167 |  |  |  | <1 | 1 | Unknown | Micro-fragments |
| 1168 |  |  |  | 3 | 1 | Mammal | Diaphysis |
| 1182 |  |  |  | <1 | 1 | Unknown | Micro-fragments |
| 2003 |  |  |  | 5 | 4 | Unknown | Micro-fragments |
| 2003 |  |  |  | 1 | 1 | Fox | Rib |

***Table 2, S9 – Macromammal specimens identified in the ledge.*** The identification number (ID) of each element recovered in the cave, and spatial coordinates when available, are provided.

1. **MICROFAUNAL ANALYSIS**

The mixed pattern of digestion and the different degrees of weathering of the small-mammal remains retrieved from the Ledge of the Horses of Atxurra Cave suggest a combined origin for the accumulation: most of it may be allochthonous, accidentally brought there within sediments (intentionally or accidentally) carried out by people from the entrance and/or the posterior passage of the cave. The remaining part may have been deposited as digestion sub-products of animals able to reach that deep in the cave (330 m from the entrance), e.g., small mammalian carnivores.

| **Scientific name** | **Common name** | **NISP** | **MNI** |
| --- | --- | --- | --- |
| *Apodemus sylvaticus* | Field mouse | 2 | 1 |
| *Arvicola sapidus* | Southwestern water vole | 10 | 3 |
| *Myodes glareolus* | Bank vole | 3 | 1 |
| *Chionomys nivalis* | Snow vole | 2 | 1 |
| *Microtus oeconomus* | Tundra vole | 4 | 2 |
| *Microtus agrestis* | Field vole | 1 | 1 |
| *Eliomys quercinus* | Garden dormouse | 1 | 1 |
| *Arvicolinae* | Arvicolines | 34 |  |
| *Rodentia* | Rodents | 2 |  |

***Table 2, S9 – Identified taxa in the microfaunal analysis.***

***S10: Virtual recreation.***

1. **SPATIAL GEOLOCATION**

Firstly, the entire cave was scanned the company GIM-Geomatics using a terrestrial Laser Scanner 3D *Faro^®^ Photon 120*. Approximately 59.6 million points have been obtained per scan, in 538 scan stations. As for the accuracy of the operation, the estimated error is 1 mm per 25 m, with 90% reflectance. From this point cloud, we have generated different three-dimensional models of the cave (one for the entire cave, other for the decorated areas with higher precision, etc.). Once all archaeological evidence in the cave (GUs and IAC elements) had been documented, they were georeferenced using a *DistoX2* device, in conjunction with a tablet using an Android system with Bluetooth, and with *TopoDroid*^®^ (Corvi, 2015) application installed (Trimmis, 2018; Intxaurbe *et al.*, 2020). Finally, all the data (geomorphology, archaeological evidence, etc.) were subsequently reintroduced into a GIS, joining it with the information stored in the databases (FileMaker^®^ and Microsoft Access^®^) with information regarding each figure (subject, technique, size, etc.), or each archaeological remains (analysis, dating, etc.). All of this was analysed spatially with ArcGIS^®^, through analysis regarding visibility (Viewshed and Lines of Sight) (Wheatley, 2004; Llobera, 2007; Landeschi *et al*., 2016; Intxaurbe *et al*., 2020; Intxaurbe *et al*., 2022), Least Cost Paths and Accessibility (Intxaurbe *et al*., 2021), Density and distribution, etc.

1. **GEOMORPHOLOGICAL RECREATION**

A geomorphological study was carried out in the entire cave (including sector J) (Arriolabengoa *et al*., 2020), because it is fundamental to determine the processes that have occurred during the evolution of a given cave and identify the different geomorphologic and sedimentological events (especially those that have altered the morphology during the Upper Magdalenian). The 3D models of the cave were subsequently modified, using *Meshlab*^®^ (to cut and work independently in areas of interest) and *Blender*^®^ (e.g., with “sculpting mode” to reduce and cut out Holocene formations). After that, the model is introduced in *ArcGIS*^®^, to create different raster-files (Scott and Janikas, 2010), following a defined procedure (Opitz and Nowlin, 2012).

1. **LIGHTING RECREATION**

The light intensity and the radius of action of the light emitted by the fireplaces and the torches has been obtained through the experimental activity, based on archaeological data (*previous phase*) obtained in the cave (woody fuel and dimensions). The experimentation was developed in a natural cave without archaeological remains (Isuntza cave) with the same geological and environmental (in particular, temperature, humidity) characteristics as Atxurra cave (Medina-Alcaide *et al*., 2021). To measure the *temperature* (ºC) of the fire and the *illuminance* (lx) we use a pyrometer (*PCE-778* model) and a lux meter (*PCE-L335* model) respectively. The measurements were repeated every 5 'and measurements were repeated every 5 'and every 10 cm from the centre of the flame in radial direction until the recording of 0 lux. The distance at which total darkness (0 lx) was registered was taken as our *radius of action* (m). More information on the methods and data on the archaeological experimentation of paleolithic light can be found in M. Medina-Alcaide et al. 2021.

The experimental torch was made up of a central nucleus of juniper branch 2 centimetres in diameter, dry and crushes (to give it greater amount of fibre, facilitating oxygenation of the combustion). Around this branch we add 75 grams of birch bark (to facilitate the start of combustion) and 5 pieces of juniper heartwood (also dry) cut tangentially (each 4 centimetres wide and 0.5 centimetres thick). All components were attached to the central shaft with ivy (green, not dry). We also include an oak handle 5 centimetres thick and 10 centimetres long. The dimensions are 47 centimetres in length, 8 centimetres maximum width and 8.13 grams of weight.

The experimental fireplace 912 g. of branches of 1-2 cm. dry juniper and oak and 75 g. of dry birch bark. The woody fuel was placed as a pyramid and the most flammable fuel was in the central core. The fire has a circular shape. Its dimensions are 23 centimetres wide and 7 cm.

| **EXPERIMENTATION** | **TORCH (Average values)** | **FIREPLACE** |
| --- | --- | --- |
| **Duration (')** | 41 | >30 |
| **Ligneous fuel (g.)** | - | 912 |
| **Illuminance (lux)** | 16.92 | 19,2 |
| **Luminous intensity (cd)** | 2.71 | 3,07 |
| **Radius (max.)** | 2,99 | 3,30 |
| **Temperature. Centre (ºC)** | 561.44 | 586,67 |
| **Temperature. Periphery (ºC)** | 112.64 | 45,00 |

***Table 1, S9 – Compilation of luminous data of experimental fire.***

1. **VIRTUAL REALITY RECREATION**

Previous studies have led to the creation of a virtual recreation of the Paleolithic art context of the "Ledge of the Horses". To achieve this, the graphics engine ©Unreal Engine 5 was used, which allows for the integration of high-quality 3D models of the cavity into a virtual environment with real-time lighting (Rodríguez *et al.,* 2023).

The original state of the cavity was virtually reconstructed with the intention of visualizing the subterranean context of rock art. To accomplish this, modern graffiti was removed, and the lighting conditions and systems were recreated based on archaeological remains found on the floor of the cornice and data resulting from experimentation. Geomorphologically, the ledge of the horses has not undergone significant modifications since the Upper Paleolithic.

By placing the fixed fires in their original position and integrating parameters such as light intensity, illuminance, radius of action or color temperature, it is possible to verify different hypotheses about the visibility of the engravings and the use of lighting systems as a scenographic element. The torch can also be manipulated with virtual reality, allowing for an immersive experience and natural movement within the virtual environment, as well as interaction with different elements of the scene such as torches or archaeological pieces. The visualization and interaction with this reconstruction is done through the virtual reality glasses ©*Meta Quest* *2* and allows, not only a way of quality scientific dissemination, but also the generation of new historical knowledge.

***S11: References in the Supplementary Information.***

Arriolabengoa, M. *et al*. From cave geomorphology to Palaeolithic human behaviour: speleogenesis, palaeoenvironmental changes and archaeological insight in the Atxurra-Armiña cave (northern Iberian Peninsula). *J. Quaternary Sci* **35**, 841-853. <https://doi.org/10.1002/jqs.3225> (2020).

Brock, F., Higham, T., Ditchfield, P. & Ramsey, C. B. Current pretreatment methods for AMS radiocarbon dating at the Oxford Radiocarbon Accelerator Unit (ORAU)”. *Radiocarbon* **52**(1), 103-112 (2010).

Bronk Ramsey, C. Dealing with Outliers and Offsets in Radiocarbon Dating. *Radiocarbon,* *51*(3), 1023-1045. <https://doi.org/10.1017/S0033822200034093> (2009).

Carrión, Y., Verdasco, C., Morales, J.V. & Aura, J.E., 2018. Au-delà du radiocarbone: analyse de taxons et contexte combinés pour la détection de problèmes taphonomiques. Un exemple dans les Grottes de Santa Maira (Alicante, Espagne). *ArcheoSciences. Rev. d’archéométrie*  **42**, 35-43 (2018).

Chabal, L., Fabre, L., Terral, J.F. & Théry-Parisot, I. L’anthracologie. In *La Botanique* (eds J.E Brochier,J.E. *et al.*) 43-105 (Errance, Paris,1999).

Corvi, M., Cave surveying with TopoDroid. In *1ª Convención Internacional Espeleología*, 59-62 (Barcelona, 2015).

González-Urquijo, J. E., & Ibáñez Estévez, J. J. Metodología de análisis funcional de instrumentos tallados en sílex. In *Cuadernos de Arqueología* 14. Universidad de Deusto (1994).

Intxaurbe, I. *et al.* Hidden images in Atxurra Cave (Northern Spain): a new proposal for visibility analyses of Palaeolithic rock art in subterranean environments. *Quaternary International*, <https://doi.org/10.1016/j.quaint.2020.04.027> (2020).

Intxaurbe, I. *et al.* Quantifying accessibility to Palaeolithic rock art: methodological proposal for the study of human transit in Atxurra Cave (Northern Spain). *Journal of Archaeological Science* **125**, 105271 (2021).

Intxaurbe, I., Garate, D., Arriolabengoa, M., & Medina-Alcaide, M. Application of Line of Sight and Potential Audience Analysis to Unravel the Spatial Organization of Palaeolithic Cave Art. *Journal of Archaeological Method and Theory* <https://doi.org/10.1007/s10816-022-09552-y> (2022).

Landeschi, G. *et al*. 3D-GIS as a platform for visual analysis: investigating a Pompeian house. *Journal Archaeological Science* **65**, 103–113 (2016).

Llobera, M. Reconstructing visual landscapes*. World Archaeology* **39**(1), 51-69 (2007).

López-Tascón, C., Pedergnana, A., Ollé, A., Rasilla, M. de la, & Mazo, C. Characterization of the use-wear and residues resulting from limestone working. Experimental approach to the parietal art of La Viña rock shelter (La Manzaneda, Asturias, Spain). *Quaternary International*. [https://doi.org/https://doi.org/10.1016/j.quaint.2020.07.012](https://doi.org/https:/doi.org/10.1016/j.quaint.2020.07.012) (2020).

Marreiros, J., Gibaja Bao, J. F., & Bicho, N. F. *Use-wear and residue analysis in archaeology*. Springer International Publishing (2015).

Medina-Alcaide, M. Á. *et al*. The conquest of the dark spaces: An experimental approach to lighting systems in Paleolithic caves. *Plos one* **16**(6), e0250497 (2021).

Nelson, M. The study of technological organization. *Journal of Archeological Method and Theory* **3**, 57–100 (1991).

Neufert, E. *Bauentwurfslehre. Grundlagen, Normen und Vorschriften über Anlage, Bau, Gestaltung, Raumbedarf und Raumbeziehungen: Maße für Gebaüude, Raüume, Einrichtungen und Geräte mit dem Menschen als Maß und Ziel.* (Berlin, 1951).

Nicosia C. & Stoops, G. *Archaeological Soil and Sediment Micromorphology*. (John Wiley & Sons Ltd, 2017).

Opitz, R. & Nowlin, J. Photogrammetric modeling þ GIS. Better methods for working with mesh data. ArcUser. <http://www.esri.com/news/arcuser/0312/files/archaeology-inventory.pdf>. (consulted 05-12-20) (2012).

Ortega, I. *et al.* L’occupation de l’Aurignacien Ancien de Barbas III (Creysse, Dorgogne): résultats préliminaires sur la fonction du site. *Paléo* **18**, 115–142 (2006).

Pastoors, A. & Weniger, G.C. Cave art in context: methods for the analysis of the spatial organization of cave sites. *Journal of Archaeological Research* **19**(4), 377–400 (2011).

Plisson, H. Examen tracéologique de quelques silex collectés sur le sol de la grotte Chauvet. *Bulletin de la Société Préhistorique Française* **102**, 145-148 (2005).

Reimer, P.J. *et al.* The IntCal20 Northern Hemisphere radiocarbon calibration curve (0-55kcal BP). *Radiocarbon* **62**, 725-757 (2020).

Ríos Garaizar, J. Organización económica de las sociedades neanderales: el caso del nivel VII de Amalda (Zestoa, Gipuzkoa). *Zephyrus* *LXV*, 15–37 (2010).

Rios-Garaizar, J. *et al.* La secuencia prehistórica de la cueva de Atxurra (Berriatua, Bizkaia): evaluación de las excavaciones de J.M. Barandiarán Ayerbe (1934-1935). *Munibe (Antropologia-Arkeologia)* ***70***, 21–34. [https://doi.org/https://doi.org/10.21630/maa.2019.70.15](https://doi.org/https:/doi.org/10.21630/maa.2019.70.15) (2019a).

Rios-Garaizar, J. *et al.* La secuencia prehistórica de la cueva de Abittaga (Amoroto, Bizkaia): evaluación de las excavaciones de J.M. Barandiarán (1964-1966). *Munibe (Antropologia-Arkeologia)* **71**. <https://doi.org/https://doi.org/10.21630/maa.2020.71.06> (2020b).

Rios-Garaizar, J. *et al.* Parietal artistic expression and discreet archeological data inside the paleolithic caves: use-wear analysis from Nerja and Etxeberri lithic assem-blages, *Arkeos* **37**, 489-494 (2015).

Rios-Garaizar, J. *et al.* Sporadic occupation in Armiña cave during the Upper Magdalenian: What for? *Journal of Archaeological Science: Reports* ***30***, 102271. <https://doi.org/10.1016/J.JASREP.2020.102271> (2020a).

Rios-Garaizar, J. *et al*. The intrusive nature of the Châtelperronian in the Iberian Peninsula. *PLoS One*, 17(3), e0265219 (2022).

Rios-Garaizar, J. Industria lítica y sociedad en la Transición del Paleolítico Medio al Superior del Cantábrico oriental: la necesidad de un enfoque integral. *Nivel Cero* **11**, 29–46 (2007).

Rios-Garaizar, J. *Industria lítica y sociedad en la Transición del Paleolítico Medio al Superior en torno al Golfo de Bizkaia*. (PUbliCan - Ediciones de la Universidad de Cantabria, 2012).

Rios-Garaizar, J. Nivel IX (Chatelperroniense) de Labeko Koba (Arrasate-Gipuzkoa): gestión de la industria lítica y función del sitio. *Munibe (Antropologia-Arkeologia)* **59**, 25–46 (2008).

Rios-Garaizar, J., & Ortega, I. Flint workshop or habitat? Technological and functional approaches towards the interpretation of site function in Bergerac region Ancient Aurignacian. In *International Conference on Use-Wear Analysis. Use-Wear 2012* (eds Marreiros, J. Bicho, N. & Gibaja J.) 162–172 (Cambridge Scholars Publishing, 2014).

Rios-Garaizar, J., de la Peña, P., & San Emeterio, A. Estudio de las industrias líticas y óseas de la cueva de Aitzbitarte III (Zona de la entrada). In *Ocupaciones humanas en la cueva de Aitzbitarte III (Renteria, País Vasco) sector Entrada: 33.000-18.000 BP(*eds Altuna, J., Mariezkurrena, K. & Rios-Garaizar, J.), 81–351. (Eusko Jaurlaritzaren Argitalpen Zerbitzu Nagusia, 2011).

Rios-Garaizar, J., Garate, D., Bourrillon, R., Gómez-Olivencia, A., & Karampaglidis, T. The Venuses Block From Arlanpe Cave (Northern Iberian Peninsula): Implications for the Origins and Dispersion of Gönnersdorf-Lalinde Style Depictions Throughout the European Magdalenian. *Oxford Journal of Archaeology* **34**(4), 321–341. <https://doi.org/10.1111/ojoa.12062> (2015).

Rios-Garaizar, J., Škrdla, P., & Demidenko, Y. E. Use-wear analysis of the lithic assemblage from LGM Mohelno-Plevovce site (southern Moravia, Czech Republic). *Comptes Rendus Palevol*, **18**(3), 353–366. <https://doi.org/10.1016/J.CRPV.2018.11.002> (2019b).

Rissetto, J.D. *Late Pleistocene Hunter-Gatherer mobility patterns and lithic exploitation in Eastern Cantabria (Spain).* Doctoral thesis. University of New Mexico, Albuquerque (2009).

Rivero, O., Ruiz, J.F., Intxaurbe, I., Salazar, S. & Garate, D. On the limits of 3D capture: A new method to approach the photogrammetric recording of Palaeolithic thin incised engravings in Atxurra Cave (northern Spain). *Digital applications in archaeology and cultural heritage* **14**, <https://doi.org/10.1016/j.daach.2019.e00106> (2019).

Rodríguez, E., Casals, J. R., & Celestino, S. Application of real-time rendering technology to archaeological heritage virtual reconstruction: the example of Casas del Turuñuelo (Guareña, Badajoz, Spain). *Virtual Archaeology Review* ***14***(28), 38-53 (2023).

Ruiz-López, J.F. *et al*. Tool mark analyses for the identification of palaeolithic art and modern graffiti. The case of Grottes d’Agneux in Rully (Saone-et-Loire, France). *Digital Applications in Archaeology and Cultural Heritage* **14**. <https://doi.org/10.1016/j.daach.2019.e00107> (2019).

Ruiz-Redondo, A. *Entre el Cantábrico y los Pirineos: el conjunto de Altxerri en el contexto de la actividad gráfica magdaleniense*. Doctoral thesis, Universidad de Cantabria. (2014).

San Emeterio Gómez, A. & Rios-Garaizar, J. Estudio de las industrias líticas de la Cueva de Aitzbitarte III (Zona del Interior). In *Aitzbitarte III (País Vasco). 26.000-13. 000 BP (Zona Profunda de La Cueva)* (eds Altuna, J., Mariezkurrena, K., Rios-Garaizar, J., San Emeterio Gómez, A.)31-186 (Eusko Jaurlaritzaren Argitalpen Zerbitzu Nagusia, Vitoria-Gasteiz) (2017).

Schweingruber, F.H. *Anatomy of European woods:* *Anatomy of European Woods: An Atlas for the Identification of European Trees, Shrubs and Dwarf Shrubs.* Paul Haupt (1990).

Scott, L. M. & Janikas, M.V. Spatial statistics in ArcGIS. In Handbook of applied spatial analysis (eds Fischer, M.M., & Getis, A) 27-41 (2010).

Stoops, G., Marcelino, V. & Mees, F. *Interpretation of Micromorphological Features of Soils and Regolith*. Elsevier (2018).

Tarriño, A. *El sílex en la cuenca vasco-cantábrica y Pirineo Navarro. Caracterización y su aprovechamiento en la Prehistoria*. (Madrid, 2006).

Trimmis, K.P. Paperless mapping and cave archaeology: A review on the application of DistoX survey method in archaeological cave sites. *Journal of Archaeological Science-Reports* **18**, 399-407 (2018).

Van der Meer, J.J.M. & Menzies, J. The micromorphology of unconsolidated sediments. *Sedimentary geology* **238**, 213-232 (2011).

Vernet, J.L., Ogereau, P., Figueral, I., Machado, C. & Uzquiano, P. *Guide d´identification des charbons de bois préhistoriques et récents. Sud-Ouest de l´Europe: France, Péninsule ibérique et Îles Canaries.* (C.N.R.S., Paris, 2001).

Wheatley, D. Making space for an archaeology of place. *Internet archaeology* **15**. https://eprints.soton.ac.uk/28800/ (consulted 05-12-2020) (2004).
